# Rhoifolin from Plumula Nelumbinis exhibits anti-cancer effects in pancreatic cancer via AKT/JNK signaling pathways

**DOI:** 10.1038/s41598-022-09581-3

**Published:** 2022-04-05

**Authors:** Bingxin Zheng, Yixin Zheng, Ningning Zhang, Yi Zhang, Baodong Zheng

**Affiliations:** 1 College of Food Science, Fujian Agriculture and Forestry University, Fuzhou, 350002 Fujian People’s Republic of China; 2 Fujian Provincial Key Laboratory of Quality Science and Processing Technology in Special Starch, Fuzhou, 350002 Fujian People’s Republic of China

**Keywords:** Proteomics, Secondary metabolism, Kinases

## Abstract

This study aimed to evaluate the anti-pancreatic cancer effects of flavonoids in Plumula Nelumbinis. High-performance liquid chromatography/quadrupole time-of-flight mass spectrometry showed that apiin, rhoifolin, and vitexin were three principal components in total flavonoids derived from Plumula Nelumbinis, with vitexin being the most abundant component. Cell viability assay revealed that apiin, rhoifolin, and vitexin could inhibit proliferation of PANC-1 and ASPC-1, with rhoifolin showing the maximum inhibitory effect. Rhoifolin inhibited cell proliferation and promoted apoptosis of pancreatic cancer cells, which was associated with up-regulated JNK and p-JNK as well as down-regulated p-AKT. Rhoifolin also inhibited cell migration and invasion, and increased the antioxidant capacity in PANC-1 and ASPC-1. Besides, AKT activator (SC79) or JNK inhibitor (SP600125) effectively reversed the anticancer effects of rhoifolin in pancreatic cancer. Quantitative proteomics analysis showed that rhoifolin altered proteomic profiles in pancreatic cancer cells. Western blot analysis showed that rhoifolin down-regulated transforming growth factor beta 2 (TGF-β2), the regulator of proteoglycan synthesis, with the concomitant down-regulation of phosphorylated SMAD family member 2 (SMAD2), the downstream effector of TGF-β2. In conclusion, rhoifolin regulates the AKT/JNK/caspase-3 and TGF-β2/SMAD2 signaling pathways, which may contribute to its anti-pancreatic cancer effects.

## Introduction

Pancreatic cancer is not sensitive to chemotherapeutic agents and, hence, is associated with high mortality and low survival rates. Therefore, the need for new drugs with limited side effects is paramount. Nelumbo nucifera Gaertn is a perennial aquatic plant with notable economic value, which is edible and has medicinal value. Previous studies have demonstrated its anti-inflammatory, antihypertensive^1^, and antioxidant^2^ functions. Plumula Nelumbinis, also known as the plumule of lotus seed, comprises of the dry young leaves and radicles between the mature seeds of the Nymphaeaceae plant. The medicinal ingredients of Plumula Nelumbinis mainly include alkaloids, flavonoids, sterols, and sterol esters^3^.

Flavonoids are bioactive ingredients present in many Chinese herbal medicines and have a wide range of physiological activities, including anticancer^4^, anti-inflammatory^5^, and antioxidant effects^6,7^. Excess free radicals in the human body cause damage to cell structure and function, and can lead to aging, cardiovascular diseases, diabetes, and cancer^8^. Flavonoids have strong antioxidant properties and can scavenge free radicals. Flavonoid monomers have been reported to exhibit antioxidant and anticancer effects^9^. For example, licoflavone has been found to attenuate the genotoxicity of cancer drugs in human peripheral lymphocytes^10^. Flavonoids can also function as in vitro enzyme inhibitors and ligands of receptors involved in signal transduction^11^. These flavonoid-protein interactions together with the antioxidant properties of flavonoids are the key features from which their potential health benefits are derived.

Transforming growth factor-β (TGF-β), a potent regulator of proteoglycan synthesis, has been implicated in many diseases including cancers^12^. Upon activation, TGF-β binds to its receptors (transforming growth factor beta receptor 1 and transforming growth factor beta receptor 2), leading to phosphorylation of a family of transcription factors named SMADs. The activated SMADs then translocate to the nucleus and regulate the transcription of TGF-β-dependent genes. The TGF-β pathway is identified as one of the 12 core signaling pathways in pancreatic cancer, and mediates either pro-tumorigenic or tumor-suppressive effects depending on the tumor stage and microenvironment^13^. Indeed, pancreatic cancer is associated with a 100% incidence of mutation in at least one gene involved in TFG-β signaling pathway. Thus, TGF-β signaling has been considered as a promising therapeutic target for pancreatic cancer.

In this study, we extracted total flavonoids from Plumula Nelumbinis. High-performance liquid chromatography/quadrupole time-of-flight mass spectrometry (HPLC-QTOF-MS) was used to analyze the monomers in total flavonoids. Monomers were used to assess the effects on PANC-1 and ASPC-1 cells’ proliferation, migration, and invasion. Quantitative proteomics analysis was used to gain insights into the mechanisms underlying the effects of monomers.

## Results

### Extraction, purification, and identification

Alcohol extraction method was employed to harvest total flavonoids from Plumula Nelumbinis. Total flavonoids (32.53 g) were extracted from 7.8 kg Plumula Nelumbinis. The extraction yield was 0.42%. The total flavonoid content in crude product, extraction product, and purified product was examined. The results suggested that total flavonoid content in the crude product was about 16.25%, that in the primary extraction product was 24.96%, and that in the final extraction product was 65.60%, indicating that total flavonoids in Plumula Nelumbinis were efficiently extracted. In addition, HPLC-Q-TOF–MS was applied to analyze the components in total flavonoids. Apiin, rhoifolin, and vitexin were confirmed as the principal components.

### In vitro simulated digestion

**Figure 1:**
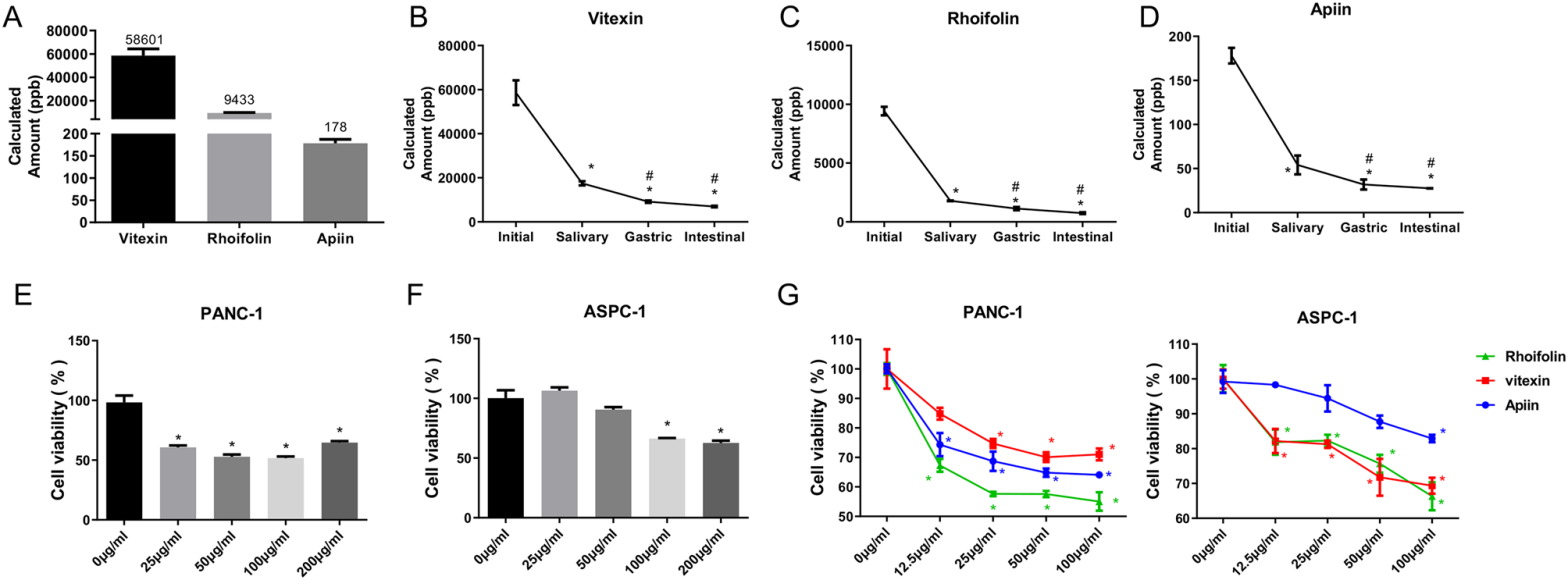
Rhoifolin might be a key effect component of total flavonoids. **(A)** The content of vitexin, rhoifolin, and apiin in the initial state. Calculated amount of vitexin **(B)**, rhoifolin **(C)**, and apiin **(D)** in plumula nelumbinis with simulated digestion of total flavonoids with salivary solution, gastric solution, and intestinal solution. Cell proliferation analysis in PANC-1 **(E)** and ASPC-1 cells **(F)** were treated with 0, 25, 50, 100, and 200 μg/ml total flavone. **(G)** Cell proliferation analysis in PANC-1 and ASPC-1 cells were processed with 0, 12.5, 25, 50, and 100 μg/ml vitexin, rhoifolin, and apiin.

Next, the standard curves with standard substances of apiin, rhoifolin, and vitexin were constructed. HPLC was used to examine the retention time (tR) of the standard substances. The results showed that the standard curve for vitexin monomer was Y = 1.075e3X + 1.194e6, R2 = 0.9843, and tR value of vitexin was 0.67 min (Fig. S1). The standard curve for rhoifolin monomer was Y = 4.895e3X – 7.415e4, R2 = 0.9989, and tR was 0.92 min (Fig. S2). The standard curve for apiin monomer was Y = 3.447e3X + 2.033e5, R2 = 0.9975, and tR was 0.98 min (Fig. S3). Based on the retention times obtained in the standard curves, the relative amounts of the three molecules in Plumula Nelumbinis could be calculated. Our data uncovered that the calculated amounts of apiin, rhoifolin, and vitexin were 178 ppb, 9433 ppb, and 58,601 ppb, respectively. Vitexin was found to be the most abundant component in total flavonoids of Plumula Nelumbinis (Fig. 1A–D). Meanwhile, in vitro simulated digestion characteristics of all three molecules with salivary solution, gastric solution, and intestinal solution were examined (Fig. S4). We found that these three solutions could effectively digest all the three major components in total flavonoid. Of the three digestive solutions, the amounts of apiin, rhoifolin, and vitexin, in total flavonoid digested by saliva were high.

### Cell proliferation study

To test whether total flavonoids might affect cell proliferation, two cell lines, including PANC-1 and ASPC-1, with five different treatment concentrations (0, 25, 50, 100, and 200 µg/ml) of total flavonoids were introduced. In the PANC-1 group, treatment with dosages of 25 µg/ml and above of total flavonoids could effectively inhibit cell proliferation compared with the control group (P < 0.05) (Fig. 1E). However, in the ASPC-1 group, only 100 µg/ml and 200 µg/ml of total flavonoids could effectively inhibit cell proliferation compared with the control group (P < 0.05) (Fig. 1F). These results indicated that the sensitivity of different pancreatic cancer cells to total polyphenol dose was different. Moreover, the inhibitory effects of different concentrations of apiin, rhoifolin, and vitexin, (0, 12.5, 25, 50, and 100 µg/ml) on the two cell lines were also assessed. In the PANC-1 cells, different concentrations of apiin, rhoifolin, and vitexin could effectively inhibit cell proliferation. It is notable that 100 µg/ml of rhoifolin had the most obvious inhibitory effect. In the ASPC-1 cells, different concentrations of apiin, rhoifolin, and vitexin could effectively inhibit cell proliferation (Fig. 1G). It is also worth mentioning that here too, 100 µg/ml of rhoifolin had the most obvious inhibitory effect. Therefore, rhoifolin was selected to perform further studies.

### Rhoifolin enhanced the antioxidant capacity of pancreatic cancer cells

**Figure 2:**
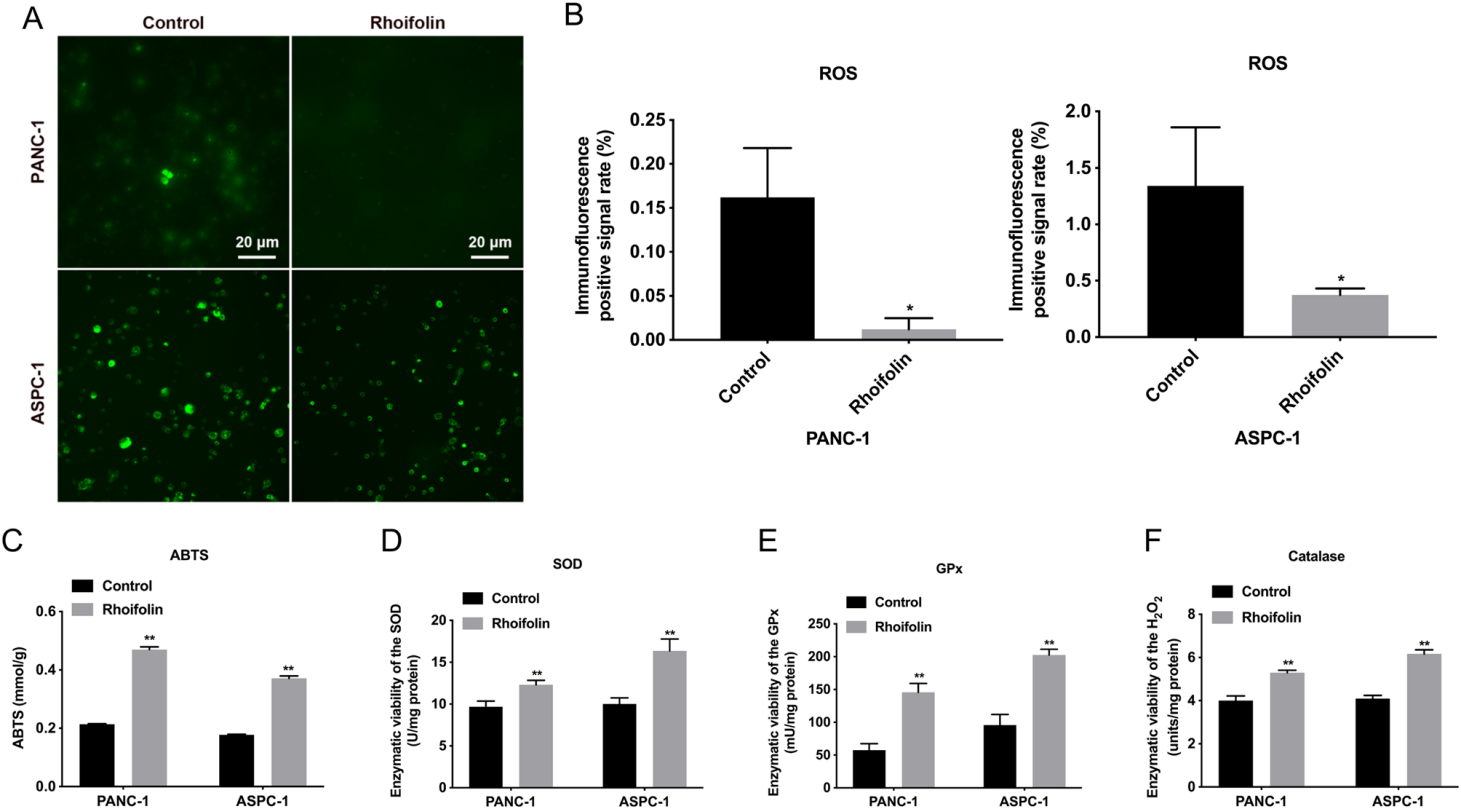
Rhoifolin enhanced the antioxidant capacity of pancreatic cancer cells. **(A)** The level of ROS was tested with ROS examination kit in PANC-1 and ASPC-1 cells disposed of rhoifolin. **(B)** And the immunofluorescence positive rate was calculated. **(C)** ABTS kit was also applied to examine antioxidant capacity of PANC-1 and ASPC-1 cells treated with or without rhoifolin. The levels of SOD **(D)**, GPx **(E)**, and catalase **(F)** were confirmed using the corresponding kits in PANC-1 and ASPC-1 cells after rhoifolin administration.

Next, we further investigated the antioxidant capacity of rhoifolin in PANC-1 and ASPC-1 cells. The reactive oxygen species (ROS) and 2,2'-azino-bis(3-ethylbenzthiazoline-6-sulfonic acid (ABTS) levels were tested to assess the antioxidant capacity of rhoifolin in PANC-1 and ASPC-1 cells. In the ROS analysis, rhoifolin treatment (100 µg/ml) of PANC-1 and ASPC-1 cells could effectively reduce the content of ROS in both cells (Fig. 2A,B). In the ABTS analysis, treatment of PANC-1 and ASPC-1 cells with rhoifolin (100 µg/ml) increased the antioxidant capacity in both cells (Fig. 2C). Besides, our data revealed that rhoifolin could cause a noteworthy elevation in superoxide dismutase (SOD), glutathione peroxidase (GPx), and catalase levels in PANC-1 and ASPC-1 cells (Fig. 2D–F). The results mentioned above suggested that rhoifolin could reduce the production of ROS and increase the antioxidant capacity.

### Rhoifolin induced the apoptosis of pancreatic cancer cells

**Figure 3:**
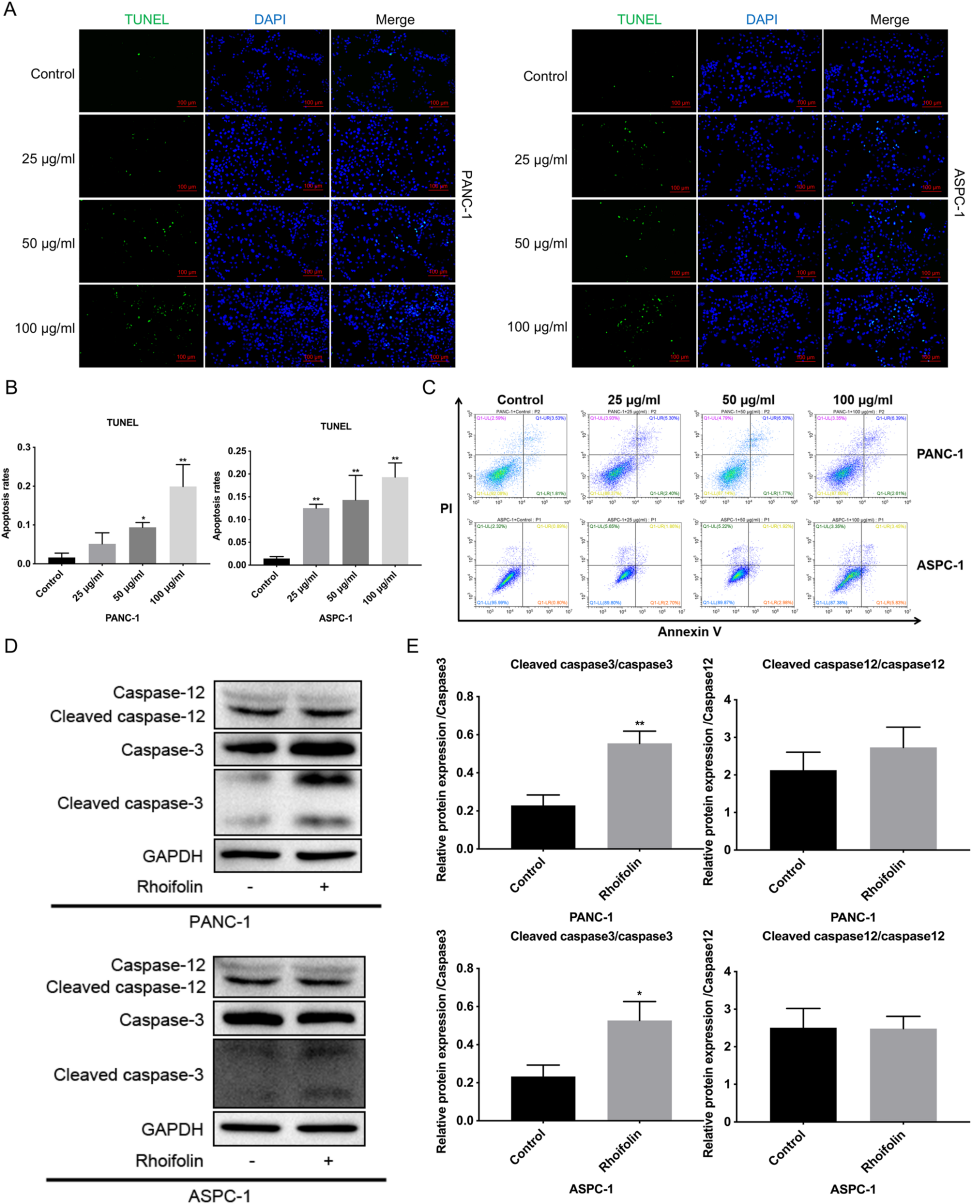
Rhoifolin induced the apoptosis of pancreatic cancer cells. PANC-1 and ASPC-1 cells were addressed with 0, 25, 50, and 100 μg/ml rhoifolin, respectively. **(A)** TUNEL positive cells were examined using TUNEL staining. **(B)** The apoptosis rate was calculated based on the results of **(A)**. (**C)** Cell apoptosis was monitored by applying Flow cytometry. **(D)** Western blotting analysis of caspase-12, Cleaved caspase-12, caspase-3, and Cleaved caspase-3 in the processed cells. **(E)** The relative comparability of Cleaved caspase-12/caspase-12 and Cleaved caspase-3/caspase-3 was counted on account of the western blotting results.

Subsequently, the anti-apoptotic role of rhoifolin was authenticated in PANC-1 and ASPC-1 cells. After treatment with 0, 25, 50, and 100 µg/ml of rhoifolin, green fluorescence was significantly increased in PANC-1 and ASPC-1 cells (Fig. 3A). In other words, rhoifolin could significantly raise the percentage of TUNEL-positive cells in PANC-1 and ASPC-1 cells (Fig. 3B). Similarly, flow cytometry data signified that cell apoptosis was increased under treatment with 100 µg/ml of rhoifolin in PANC-1 and ASPC-1 cells (Fig. 3C). Caspase-12 and caspase-3 are the apoptotic marker proteins. Western blot results denoted that cleaved caspase-3/caspase-3 ratio was significantly higher in the rhoifolin treatment group than that in the control group (Fig. 3D,E). However, there were no significant changes between the two groups (Fig. 3D,E). On the whole, we concluded that rhoifolin could promote pancreatic cancer cell apoptosis, which might be relevant to cleaved caspase-3.

### Rhoifolin prevented the migration and invasion, and regulated AKT/JNK pathway in pancreatic cancer cells

**Figure 4:**
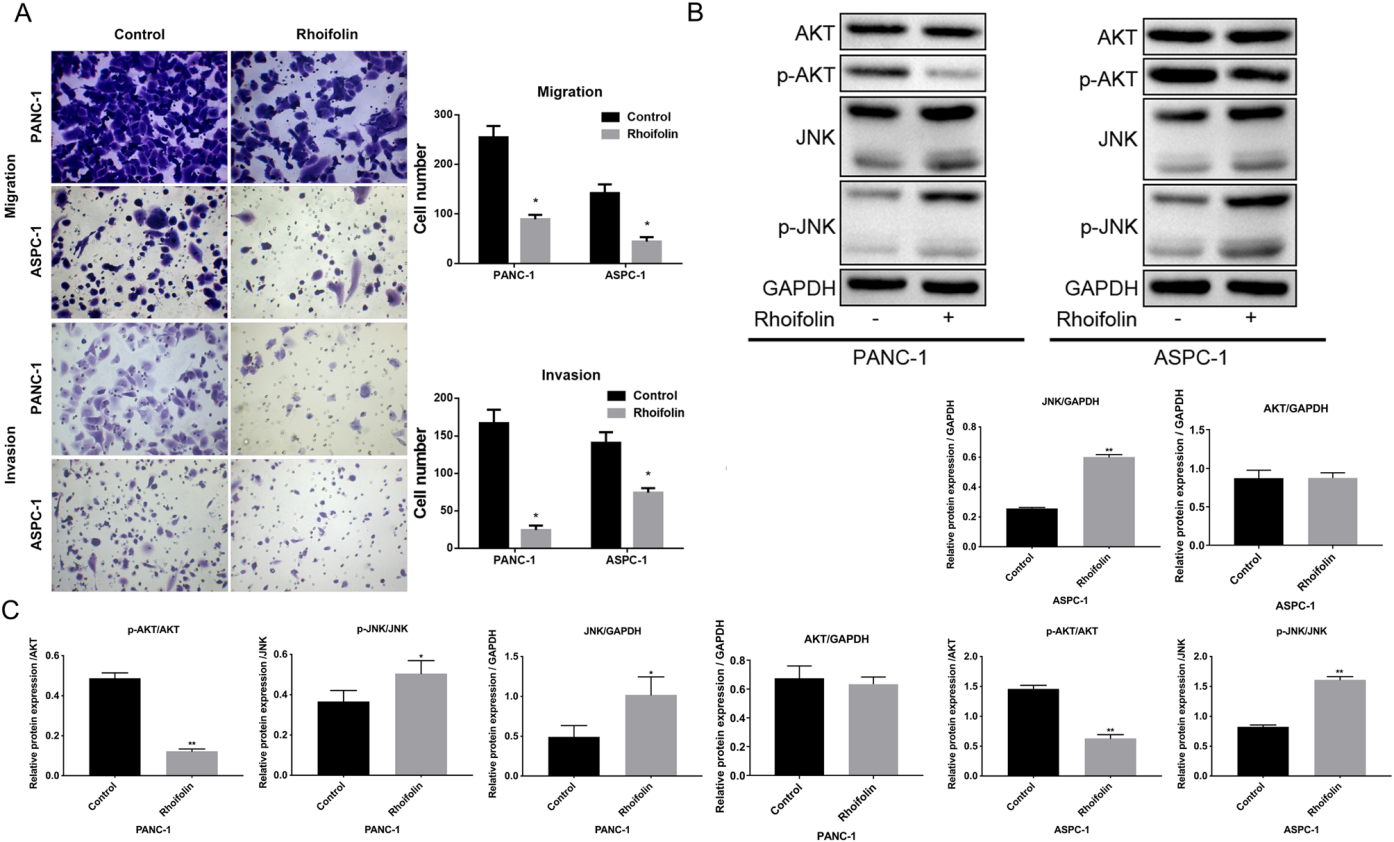
Rhoifolin prevented the migration and invasion, and regulated AKT/JNK pathway in pancreatic cancer cells. PANC-1 cells or ASPC-1 cells were dealt with 100 µg/ml rhoifolin. **(A)** Transwell was utilized to determine the migration and invasion. **(B)** JNK, p-JNK, AKT, and p-AKT expressions were analyzed via western blotting analysis in the processed PANC-1 and ASPC-1 cells. **(C)** Quantitative analysis of p-AKT/AKT, AKT/GAPDH, p-JNK/JNK and JNK/GAPDH.

In order to investigate the effects of rhoifolin on the in vitro migration and invasion potential of tumor cells (PANC-1 and ASPC-1 cells), transwell chambers were applied. The cell migration analysis showed that the number of PANC-1 and ASPC-1 cells that migrated on treatment with rhoifolin was significantly lower than that in the control group (Fig. 4A). These results suggested that rhoifolin could effectively inhibit cell migration. Meanwhile, the cell invasion analysis revealed that the extent of invasion was also significantly reduced in the rhoifolin-treated PANC-1 and ASPC-1 cells compared with the control group (Fig. 4A). The results showed that rhoifolin treatment could effectively suppress cell invasion. c-Jun N-terminal kinase (JNK), also known as stress-activated protein kinase, is an important member of the mitogen-activated protein kinase (MAPK) family, and plays a regulatory role in cell apoptosis^14,15^. Western blot data showed that the values of p-JNK/JNK and JNK/GAPDH were significantly higher in rhoifolin-treated PANC-1 and ASPC-1 cells than that in the control group, indicating that rhoifolin could induce the JNK pathway in the apoptosis of pancreatic cancer cells. JNK is one of the essential downstream signaling pathways of AKT (protein kinase B). Although the value of AKT/GAPDH in PANC-1 and ASPC-1 cells was insignificantly changed after treatment with rhoifolin, the value of p-AKT/AKT in rhoifolin-treated cells was significantly lower than that in the control group. This indicated that rhoifolin could prevent the phosphorylation of AKT (Fig. 4B,C). Based on the above results, it could be surmised that rhoifolin regulates cell proliferation and apoptosis through the AKT/JNK signaling pathway. Therefore, rhoifolin could be utilized as a potential anticancer drug.

### AKT activator or JNK inhibitor could reverse the enhanced antioxidant capacity of rhoifolin in pancreatic cancer cells

**Figure 5:**
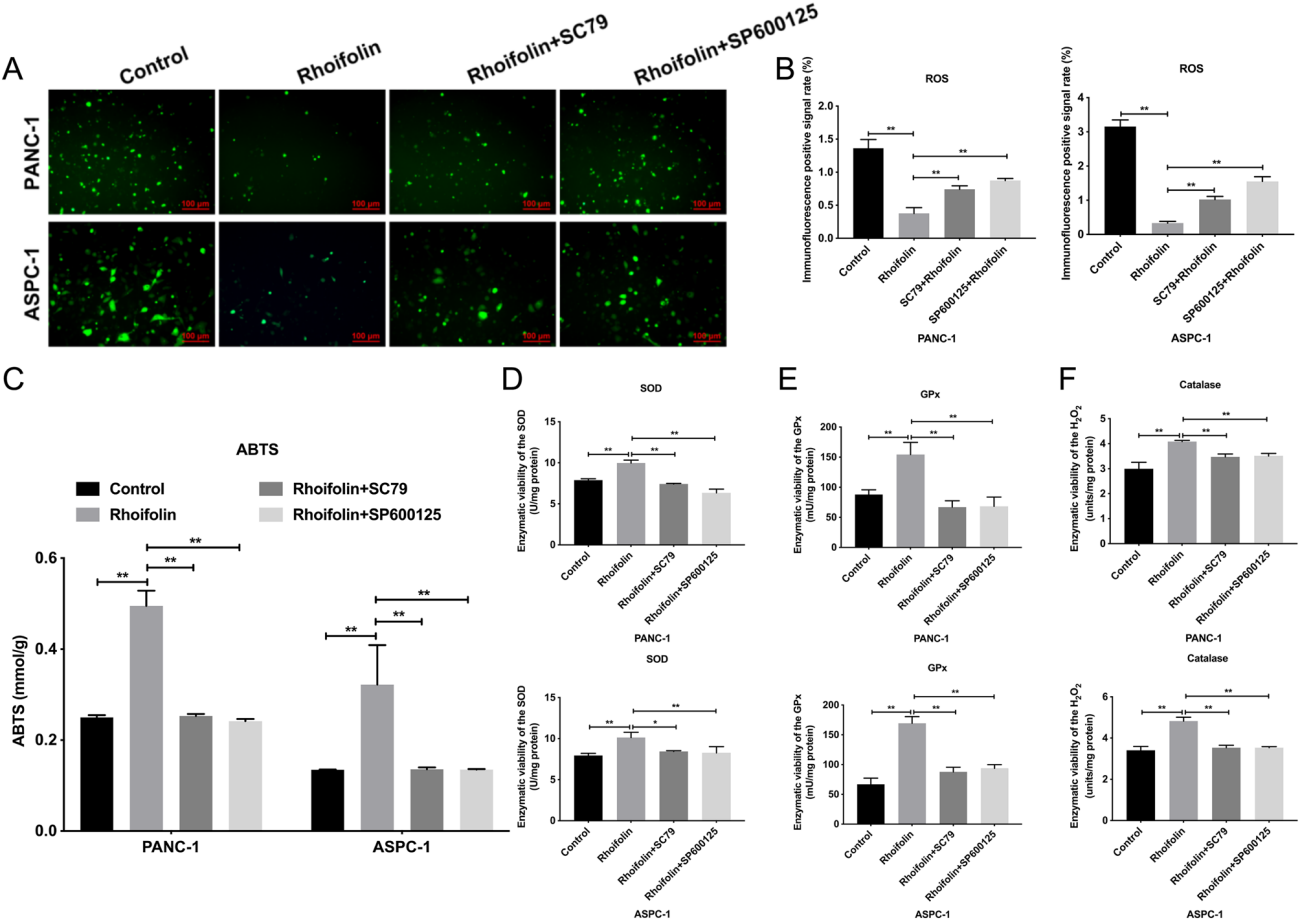
AKT activator or JNK inhibitor could reverse the enhanced antioxidant capacity of Rhoifolin in pancreatic cancer cells. Rhoifolin-treated PANC-1 and ASPC-1 cells were also addressed with AKT activator (SC79) or JNK inhibitor (SP600125). **(A)** ROS level was assessed using DCFH-DA in ROS examination kit. **(B)** The positive rate of ROS in **(A)** was quantified. **(C)** The antioxidant capacity was evaluated using the ABTS kit. SOD **(D)**, GPx **(E)**, and catalase **(F)** levels were monitored by applying the corresponding kits.

Based on our findings of suppression of pancreatic cancer, and inhibition of AKT pathway and induction of JNK pathway mediated by rhoifolin, we further verified whether rhoifolin could influence pancreatic cancer progression by changing the expression of AKT or JNK pathways. The results revealed that rhoifolin markedly reduced the level of ROS; however, the decreased ROS level induced by rhoifolin was reversed by AKT activator (SC79) or JNK inhibitor (SP600125) in PANC-1 and ASPC-1 cells (Fig. 5A,B). We also discovered that rhoifolin observably strengthened the ABTS concentration in PANC-1 and ASPC-1 cells; however, the increase in ABTS concentration induced by rhoifolin could be restrained by SC79 or SP600125 (Fig. 5C). Besides, we also noted that rhoifolin dramatically elevated the levels of SOD, GPx, and catalase in pancreatic cancer cells; however, elevated levels of SOD, GPx, and catalase induced by rhoifolin also could be significantly attenuated by SC79 or SP600125 (Fig. 5D–F). On the whole, our data uncovered that the antioxidant capacity of rhoifolin was induced in pancreatic cancer cells through AKT and JNK pathways.

### Rhoifolin suppressed proliferation, migration, and invasion, and accelerated apoptosis by JNK and AKT pathways in pancreatic cancer cells

**Figure 6:**
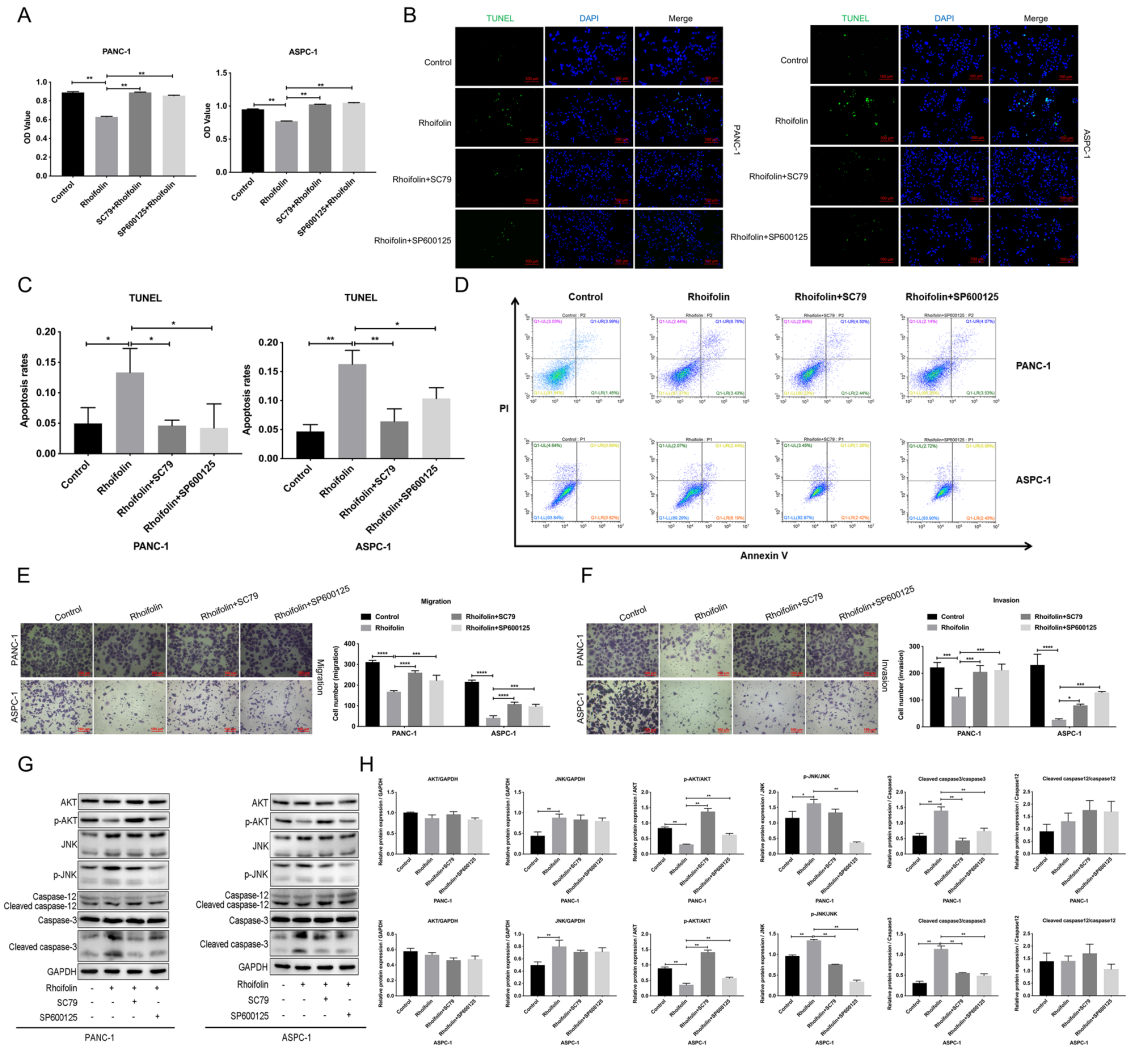
Rhoifolin suppressed proliferation, migration and invasion, and accelerated apoptosis by JNK and AKT pathways in pancreatic cancer cells. PANC-1 and ASPC-1 cells were dealt with rhoifolin, SC79, or/and SP600125, respectively. **(A)** Cell proliferation analysis of PANC-1 and ASPC-1 cells after processing. **(B)** Cell apoptosis was tested with TUNEL staining. **(C)** The rate of TUNEL positive cells was calculated. **(D)** Flow cytometry was utilized to monitor cell apoptosis. (**E,F**) The migrated and invaded cells were assessed with Transwell. (**G**) Western blotting analysis of AKT, p-AKT, JNK, p-JNK and apoptosis-related proteins in the processed cells. (**H**) Relative quantitative analysis of the western blotting results.

Furthermore, we also conducted a series of experiments to test whether AKT/JNK pathways participate in the influence of rhoifolin on the proliferation and apoptosis of pancreatic cancer cells. As displayed in Fig. 6A, rhoifolin treatment caused a striking reduction in the proliferation of PANC-1 and ASPC-1 cells; however, the reduced effect on cell proliferation induced by rhoifolin was weakened by SC79 or SP600125 (Fig. 6A). Additionally, TUNEL staining and flow cytometry results exhibited that SC79 or SP600125 notably attenuated the enhancement of cell apoptosis mediated by rhoifolin in PANC-1 and ASPC-1 cells (Fig. 6B–D). Meanwhile, transwell results indicated that the decrease in cell migration and invasion mediated by rhoifolin could also be reversed by SC79 or SP600125 in PANC-1 and ASPC-1 cells (Fig. 6E,F). Moreover, rhoifolin prominently increased the values of JNK/GAPDH, p-JNK/JNK, and cleaved caspase-3/caspase-3, and decreased the value of p-AKT/AKT in PANC-1 and ASPC-1 cells, these changes in values mediated by rhoifolin could be dramatically reversed by SC79 or SP600125 (Fig. 6G,H). On the whole, these findings show that rhoifolin could attenuate the process of tumorigenesis in pancreatic cancer cells through JNK and AKT pathways.

### Identification of differentially expressed proteins (DEPs) in pancreatic cancer cells after rhoifolin treatment

**Figure 7:**
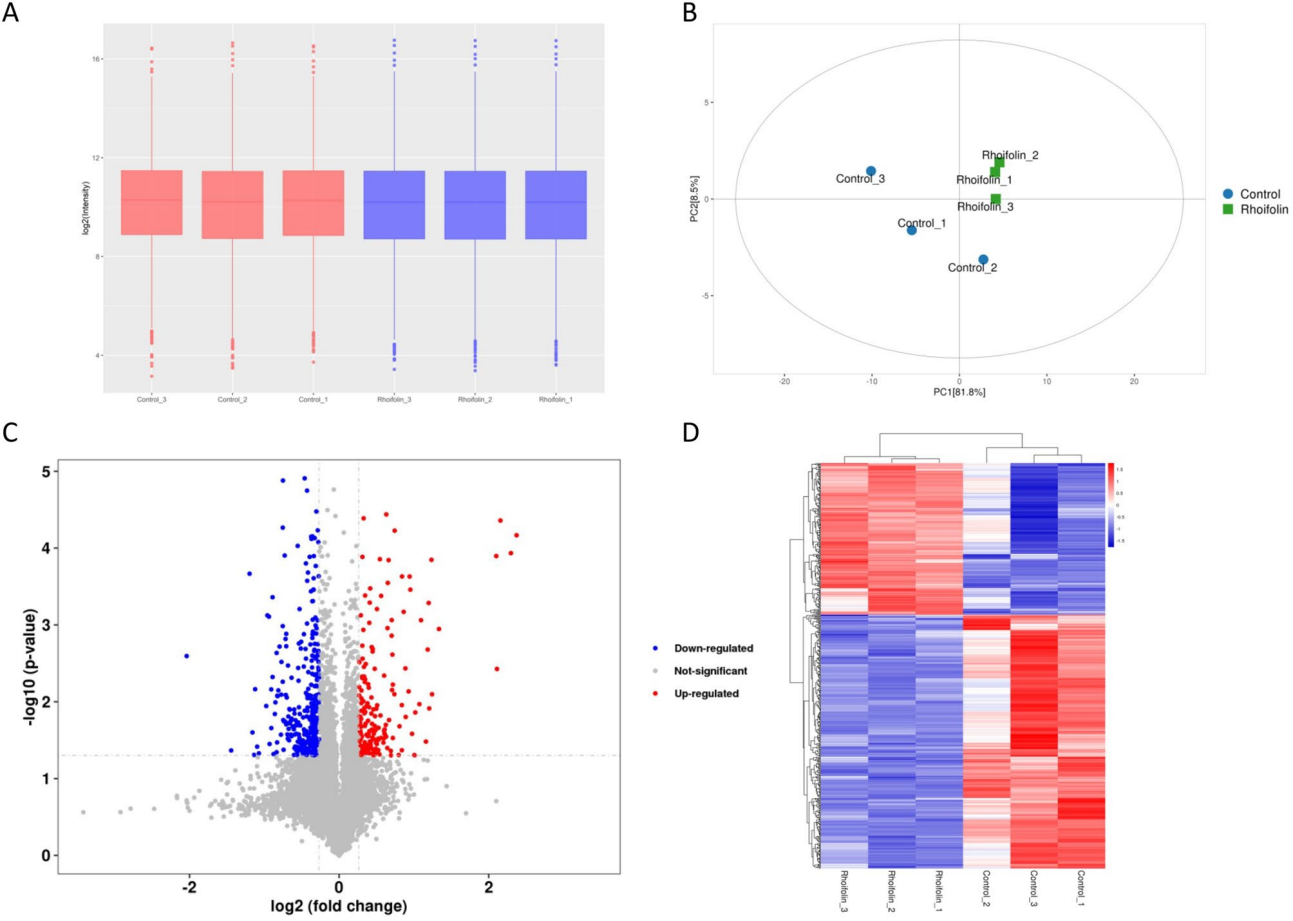
Identification of DEPs by rhoifolin treatment. (**A**) The box plot of log2 of the protein intensity in each sample. (**B**) The principal component analysis (PCA) of all 6 samples. (**C**) The volcano plots of 522 DEPs, Thresholds:fold change > 1.2 or < 0.83; Student’s t-test: p value < 0.05. (**D**) The heatmap of hierarchical clustering analysis of 522 DEPs. Rows represent proteins, and columns represent samples.

To further investigate the mechanisms underlying the toxic effects of rhoifolin on pancreatic cancer cells, we performed TMT-based quantitative proteomics analysis based on nanoLC-MS/MS. A total of 81,123 unique peptides mapping to 8,474 unique proteins were detected. The median of the log2 ratios of protein intensities were close for all samples, suggesting no obvious bias between various LC–MS/MS runs (Fig. 7A). Principal component analysis analysis showed that three rhoifolin-treated cell samples gathered together and separated from the control samples (Fig. 7B). A total of 522 DEPs between the rhoifolin-treated group and the control group were identified by statistical analysis (Student’s t-test, P < 0.05, fold change [FC] > 1.2 or < 0.83) (Fig. 7C). Hierarchical clustering analysis revealed that the rhoifolin-treated group had a significantly different proteomic expression profile compared with the control group, with 195 DEPs up-regulated and 327 DEPs down-regulated after rhoifolin treatment (Fig. 7D). These results suggest that rhoifolin markedly alters the proteomic pattern in PANC-1 cells.

### Bioinformatics analysis of DEPs in pancreatic cancer cells after rhoifolin treatment

**Figure 8:**
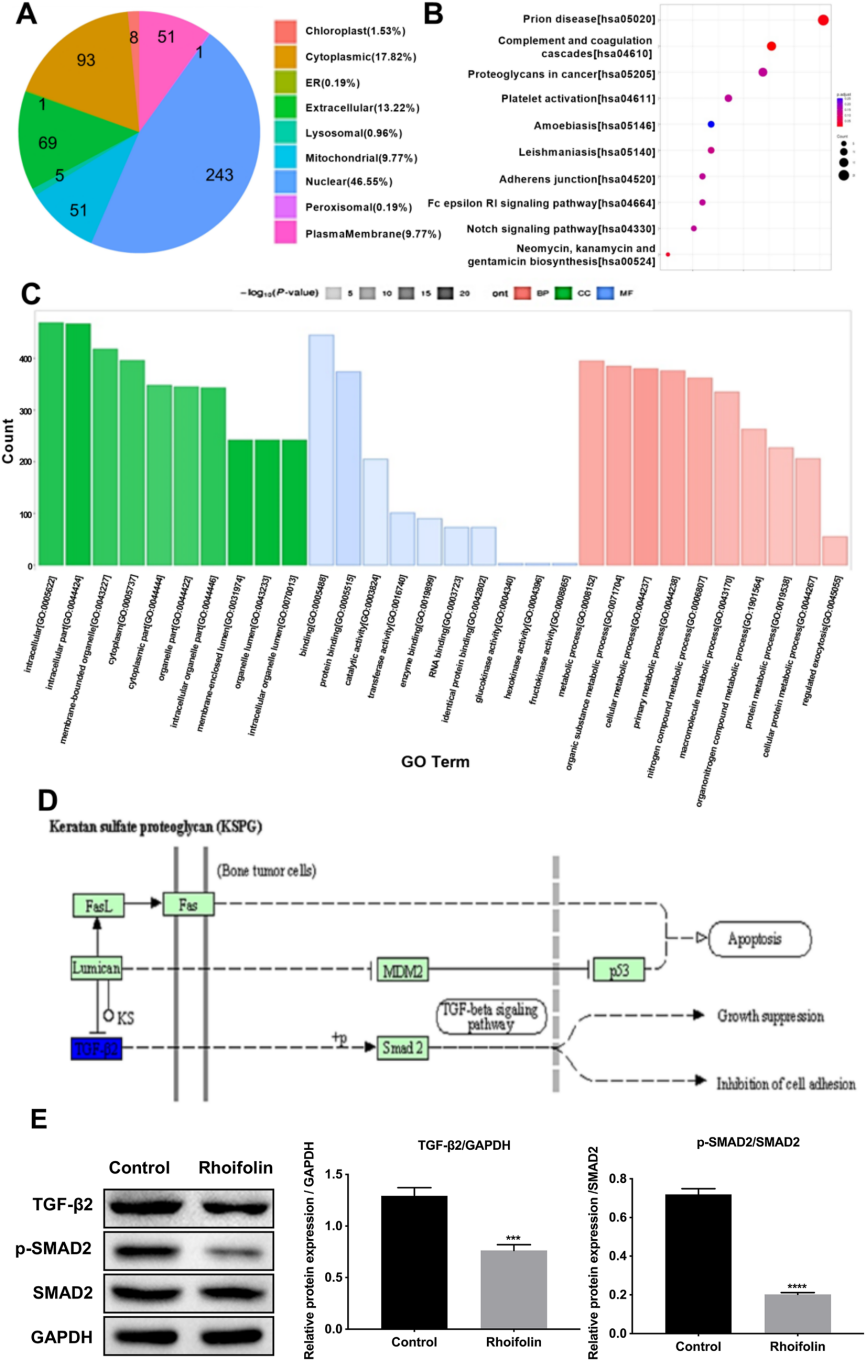
Bioinformatics analysis of DEPs. All 522 DEPs underwent the analysis of subcellular localization (**A**), the enrichment analysis of KEGG signaling pathways (**B**) and Gene Ontology annotation (**C**). (**D**) The DEPs in the “Proteoglycans in cancer” KEGG pathway. (**E**) Western blot analysis of TGF-β2, SMAD2, and p-SMAD2 in PANC-1 cells treated with or without rhoifolin. GAPDH was used as internal reference.

As shown in Fig. 8A, proteomics analysis of subcellular localization showed that after rhoifolin treatment, about 50% of the DEPs were localized in the nucleus, thereby suggesting that rhoifolin targets mainly nuclear proteins. To gain insights into the functions of the DEPs induced by rhoifolin treatment, we performed both the Kyoto Encyclopedia of Genes and Genomes (KEGG) pathway enrichment analysis and gene ontology (GO) annotation enrichment analysis. The top 10 most enriched and KEGG pathways are shown in Fig. 8B. GO annotation analysis revealed that the DEPs induced by rhoifolin were mostly enriched in “Molecular Function” and “Biological Process” (Fig. 8C). It should be noted that the “Proteoglycans in cancer” pathway was among the top three most significantly enriched KEGG pathways. Given the importance of proteoglycans in cancer metastasis, we further evaluated the effect of rhoifolin on the expression of proteins in this pathway, and demonstrated that TGF-β2, a key regulator of proteoglycan synthesis, was significantly down-regulated by rhoifolin (Fig. 8D). Activation of TGF-β2 leads to phosphorylation of SMAD2. Western blot assay showed that rhoifolin significantly decreased the protein levels of both TGF-β2 and phosphorylated SMAD2 in PANC-1 cells (Fig. 8E). These results suggest that rhoifolin alters proteoglycans in pancreatic cancer cells through inhibition of TGF-β2/SMAD2 pathway.

## Discussion

Pancreatic cancer is a digestive system tumor whose incidence is increasing every year globally. In China, the incidence of pancreatic cancer has increased by about six times in the past 20 years, and the mortality rate ranks fifth among malignant tumors^16^. Currently, there are no tumor markers with sufficient sensitivity and specificity for screening and diagnosis of early pancreatic cancer. Surgical resection is still the mainstay of treatment in pancreatic cancer in clinical practice; this is supplemented by chemotherapy, radiotherapy, physical therapy, and biological therapy. In the field of biotherapy, the use of monoclonal antibodies against vascular endothelial growth factor is still in clinical trials. Surgical resection combined with chemotherapy is the more common treatment modality in pancreatic cancer. However, total surgical resection rate and 5-year survival rate of pancreatic cancer have not increased significantly in the past 20 years^17^. Pancreatic cancer is less sensitive to chemotherapeutic drugs. Moreover, chemotherapeutic drugs are harmful to normal human tissues. Therefore, the detailed molecular mechanism of pancreatic cancer needs to be elucidated so that effective treatment methods, and sensitive drugs with limited side effects can be devised and this is currently the focus of cancer research^18^.

Flavonoids are natural bioactive phenolic compounds that could be valuable anticancer drugs^19^. Incidence of pancreatic cancer in smokers who eat foods containing more flavonols in their daily lives was significantly reduced^20^. Rossi et al. reported that proanthocyanidins could significantly reduce the risk of developing pancreatic cancer; flavanols, flavanones, flavonols, anthocyanins, flavonoids, monomeric proanthocyanidins, and dimeric proanthocyanidins protect against pancreatic cancer^21^. Several flavonoids, including apigenin, quercetin, luteolin, and vitexin^21–25^, have been shown to inhibit the proliferation of pancreatic cancer cells. Their inhibitory mechanisms have also been extensively investigated. For instance, apigenin can separate P53/Bcl-xl to allow the nuclear translocation of P53, which promotes the expression of PUMA and P21, leading to the alterations in mitochondrial permeability, release of cytochrome c, and induction of apoptosis^26^. In addition, apigenin downregulates cycB expression, leading to blockage of cell mitosis and G2/M phase arrest ^22^. In this study, total flavonoids were extracted from Plumula Nelumbinis. HPLC-Q-TOF–MS was applied to analyze the main components, including apiin, rhoifolin, and vitexin, in the total flavonoids from Plumula Nelumbinis. Rhoifolin was identified as the component that most effectively inhibited PANC-1 and ASPC-1 cell proliferation. Our functional studies have for the first time shown that rhoifolin has multiple roles in suppressing cancer cell development, including inducing cell apoptosis, oxidation resistance, and inhibition of proliferation, migration, and invasion.

AKT is a serine/threonine protein kinase that can phosphorylate several proteins and itself, playing an important role in regulating cell survival and other multiple functions^27,28^. AKT is also a key molecule in the PI3K/AKT signaling pathway^28^. Its phosphorylation function can activate different substrates, which could promote tumor cell growth, invasion, metastasis, and inhibit tumors cell apoptosis^29^. Multiple studies have confirmed that targeting the AKT pathway could be regarded as a key strategy in the treatment of pancreatic cancer^30,31^. Activation of AKT phosphorylation causes the progression of pancreatic cancer^30–32^. JNK is a member of the MAPK family and is a serine/threonine protein kinase^33^. In cancer cells, the JNK signaling pathway has been shown to play an important role in regulating apoptosis, enhancing tumor resistance, promoting cell proliferation, invasion, and metastasis^34^. Caspase-3 is considered to be an apoptotic executor, which mediates apoptosis in various tumor cells in several ways, including degradation of anti-apoptotic proteins and cleavage of damaged DNA^35^. Activation of caspase-3 is a key factor in the process of apoptosis^36^. Several studies have also confirmed that the JNK pathway underpins the biological processes of pancreatic cancer^37–39^. In the present study, we showed that rhoifolin up-regulated the expressions of JNK and p-JNK, but down-regulated p-AKT in PANC-1 cells and ASPC-1 cells. In addition, rhoifolin up-regulated the expression of caspase-3, but not caspase-12 in PANC-1 cells and ASPC-1 cells. Therefore, AKT/JNK/caspase-3 pathway appears to be involved in rhoifolin-induced apoptosis in pancreatic cancer cells. Moreover, we definitively demonstrated that AKT activator (SC79) or JNK inhibitor (SP600125) could reverse the anticancer effects of rhoifolin on pancreatic cancer cells, including enhancement of antioxidant capacity, inhibition of proliferation, migration and invasion, and induction of apoptosis. Thus, we showed that rhoifolin mediated its inhibitory effects on pancreatic cancer cells through the AKT/JNK pathway.

Proteoglycans are key molecular effectors of cell surfaces and extracellular matrix (ECM), and play a crucial role in cancer progression, invasion, and metastasis. The aberrant expression of specific proteoglycans has been associated with the progression and poor prognosis of pancreatic cancer^40^. In the present study, we showed that rhoifolin significantly altered the proteomic pattern in pancreatic cells, particularly the proteins involved in the “Proteoglycans in cancer” KEGG pathway. TGF-β is a key regulator of proteoglycan synthesis. In addition, TGF-β deregulation is involved in the pathophysiology of pancreatic cancer. It should be noted that TGF-β plays a dual role in pancreatic cancer. TGF-β shows tumor-suppressive effects in early stages of pancreatic cancer by promoting apoptosis and inhibiting the cell cycle. However, TGF-β promotes tumor neoangiogenesis and metastasis in later stages of pancreatic cancer^41^. Our results demonstrated that rhoifolin significantly inhibited the TGF-β2/SMAD2 signaling pathway in pancreatic cells, which may contribute to its inhibitory effect on cancer cell migration and invasion. However, it is not completely clear whether rhoifolin blocks tumor angiogenesis and metastasis in pancreatic cancer through the TGF-β2/SMAD2 pathway. This is a limitation of our study; future research done by us may perhaps shed light on this matter.

## Materials and methods

### Plant material and extract preparation

Fresh Plumula Nelumbinis was obtained from Lutian company (Fujian, China). The Plumula Nelumbinis was first dried in a cabinet oven with air circulation at 40 °C for 48 h and then was ground to powder using a laboratory mill. Total flavonoids of Plumula Nelumbinis were extracted with alcohol, as described previously^42^. Dried Plumula Nelumbinis (13 kg) was subjected to extraction three times with 50% ethanol in a ratio of 1:10 for 2 h each time. The extracts were combined and concentrated under reduced pressure to obtain a concentrated solution with a density of about 1.2 g/ml. Subsequently, the concentrate was extracted with ethyl acetate as reported previously^43^. Moreover, the extract was further purified by polyamide column chromatography (02395-250G, Sigma, Missouri, USA). The purified liquid was concentrated into an extract. The total flavonoids in extracts were determined according to the method used by Ismail et al.^44^. An aliquot (0.1 ml) of each extract was added to 0.3 ml 5% (w/v) NaNO_2_ and incubated for 5 min. Then, 0.3 ml 10% (w/v) AlCl_3_ and 2 ml 1 mol/l NaOH were added and the total volume was made up to 5 ml with distilled water. The total flavonoid concentration was determined by an ultraviolet spectrophotometer (NanoDrop 3000, Thermo fisher, Massachusetts, USA).

### HPLC-QTOF–MS

Agilent 1260 HPLC with G1315DAD detector, Gl312B binary pump, G1316B column oven, G1367D autosampler, and G6520B Q-TOF–MS monitor was used to detect flavonoids. For spectral analysis, the detailed parameters were: ZORBAX C18 chromatographic column was 2.1 × 100 mm and 1.8 µm; mobile phase A was acetonitrile; mobile phase B was ultrapure water. The gradient elution procedure was as follows: 0 min, 5% A; 20–22 min, 95% A; 22.1 min 5% A; 22.1–28 min, 5% A. The flow rate was 0.3 ml/min. The injection volume was 2 μl. For MS analysis, the detailed parameters were as follows: dual electros-pray ionization was employed to detect flavonoids in Plumula Nelumbinis; the capillary voltage was 3.5 kV; the atomizer pressure was 40 psi; the drying gas flow rate was 10 l/min; the drying gas temperature was 350 °C; the collision gas was high purity N2. Data were searched for the qualitative research. Standard substances of rhoifolin (B21484-20, YuanYE Biotechnology, Shanghai, China), apiin (B20980-10, YuanYE Biotechnology), and vitexin (A506797-0020, Sangon Biotechnology, Shanghai, China) were used to construct standard curves.

### In vitro simulated digestion

For the preparation of saliva, the method applied by Urszula et al.^17^ was used. The saliva preparation process was as follows: 2.38 g Na_2_HPO_4_, 0.19 g KH_2_PO_4_, 8 g NaCl, and 0.91 g α-amylase (220 U/ml) (Sigma, USA) were dissolved in 1 l of water. The pH of the solution was adjusted to 6.75 with phosphate buffer solution. Five milligrams substance was added in a test tube. Prepared saliva digestive solution (5 ml) was added and mixed. The digested solution was shaken at 37 °C for 10 min in a 100 rpm/min water bath. The digestive solution (1 l) was taken as an oral digestion group and stored at –80 °C. For the preparation of gastric juice, we referred to the method applied by Hou et al.^18^. An amount of 4 ml of 9 mg/ml NaCl solution and 4 ml of 4 mg/ml pepsin solution (Sigma, USA) were added to the 10 ml colorimetric tube and mixed. An amount of 0.1 mol/l HCl (Sigma, USA) was adjusted to a pH of 2.0. An aliquot of 1 ml of oral digestive solution was shaken at 37 °C, at 100 rpm/min in a water bath for 2 h. After 120 min of gastric digestion, the digestive juice was used as the gastric digestive group. For the preparation of intestinal juice digestion, the pH value of the above gastric juice was adjusted to 6.5–7.0 with 0.1 mol/l NaHCO_3_ solution; the mixture was shaken at 37 °C, at 100 rpm/min in a water bath for 45 min; 1.8 ml pancreatic juice-bile mixture was added to adjust the pH value to 7.5 for intestinal juice digestion. The digestive solution, after 120 min of intestinal digestion, was used as the intestinal digestive group. All experiments were conducted in parallel three times.

### Cell treatment

PANC-1 and ASPC-1 cells were purchased from ATCC (Virginia, USA), and maintained in RPMI 1640 with 10% (v/v) fetal bovine serum (FBS) (Invitrogen, Carlsbad, CA, USA). Cell lines were maintained in a humidified chamber at 5% CO_2_ at 37 °C. Firstly, PANC-1 and ASPC-1 cells were treated with 0, 25, 50, 100, and 200 μg/ml total flavone. Secondly, PANC-1 and ASPC-1 cells were processed with 0, 12.5, 25, 50, and 100 μg/ml vitexin, rhoifolin, and apiin. Thirdly, PANC-1 and ASPC-1 cells were administrated 100 µg/ml rhoifolin, 4 μg/ml AKT activator (SC79; MCE, cat. no. HY-18749), or/and 10 μM JNK inhibitor (SP600125; MCE, cat. no. HY-12041).

### MTT

The processed PANC-1 and ASPC-1 cells were evenly seeded into 96-well plates with 5 × 10^3^ cells/well, and then MTT solution (Sigma) was tested after 48 h. Each well was inoculated with 20 μl of 5 mg/ml MTT. The culture medium was discarded after 4 h culture, 150 μl DMSO was added to each well. After shaking for 10 min, the absorbance value was measured by a microplate reader and the proliferation activity of the cells was compared.

### TUNEL analysis

Cell apoptosis was examined with commercial TUNEL kit (C1062M, Beyotime Biotechnology). The detailed operations followed were as provided by the manufacturer and a previous study^45^.

### Flow cytometry

Cell apoptosis was tested using Annexin V-FITC/PI kit (BD, Cat. No.556547). Briefly, the treated PANC-1 and ASPC-1 cells were harvested and suspended in PBS. Then, cells were stained with 5 μl Annexin V-FITC and 5 μl PI for 10 min after centrifugation and suspension in 500 μl of 1 × binding buffer. Cell cycle distribution and apoptosis rate were confirmed using a flow cytometer (BD Biosciences, USA).

### Migration and invasion assay

Cell migration assay was carried out using the Transwell Permeable Support (Corning Incorporated, Corning, NY, USA). PANC-1 and ASPC-1 cells were carefully transferred on the top chamber of each transwell apparatus at a density of 1 × 10^6^ per ml with 100 µl per chamber. Cells were allowed to migrate for 24 h at 37 °C. Cells that had penetrated to the bottom side of the membrane were then fixed in methanol, stained using hematoxylin, and counted under a microscope. Cell invasion was analyzed by using the Cultrex 24-well BME Cell Invasion Assay (Trevigen Inc., Gaithersburg, MD, USA) according to standard procedures. Briefly, 1 × 10^3^ cells were seeded in 100 µl serum-free media into the upper wells previously coated with matrigel basement extract, and 500 µl of media was added to the bottom wells. After 24 h of incubation with CO_2_ at 37 °C, the non-invasive cells on the upper surface were removed and the cells that had migrated to the lower surface were fixed in 500 µl of Cell Dissociation Solution Calcein-AM, and incubation done at 37 °C in a CO_2_ incubator for 1 h; quantification was done by fluorimetric analysis (485 nm excitation, 520 nm emission).

### Antioxidant ability

Commercial ROS examination kit (S0033) and ABTS examination kit (S0119) were obtained from Beyotime Biotechnology (China). The levels of SOD, GPx, and catalase were tested using SOD activity detection kit (Beyotime, China, Cat. no. S0101S), Total Glutathione Peroxidase Assay Kit with NADPH (Beyotime, China, Cat. no. S0058), and Catalase Assay Kit (Beyotime, China, Cat. no. S0051), respectively. All operational steps were carried out as per the manufacturer’s recommendations.

### Quantitative proteomics

Sample preparation was done as follows. Cells were mixed with 200 μl of RIPA lysis buffer, and put into an ice bath under ultrasonication for 2 min. The mixtures were centrifuged at 12,000 rpm for 10 min at 4 °C. The supernatants were collected, and protein concentrations were quantified using bicinchoninic acid (BCA) protein assay. Proteins (100 μg) at 1 mg/ml concentration from each sample were mixed with five volumes of pre-chilled (–20 °C) acetone for protein precipitation overnight at –20 °C. The mixtures were then centrifuged at 12,000 rpm for 10 min at 4 °C to discard the supernatants. The pellets were washed with 200 μl of 80% pre-chilled (–20 °C) acetone twice, and centrifuged at 12,000 rpm to discard the supernatants. Next, the pellets were re-dissolved in 100 μl of 100 mM HEPES containing 1% sodium deoxycholate (SDC) by sonication for 5 min. Proteins were reduced with 5 mM (final concentration) DTT for 10 min at 55 °C, and alkylated with 10 mM iodoacetamide (IAA) for 15 min in dark at room temperature (RT). The proteins were digested with 0.5 μg/μl trypsin and incubated overnight at 37 °C. Peptide samples were collected by centrifugation and labeled with TMT (Thermo Scientific, USA) according to the manufacturer’s protocol. After SDC was precipitated by trifluoroacetic acid (TFA) and removed by centrifugation, the peptide samples were desalted on a C18 solid-phase extraction column (3 M Empore, USA).

NanoLC-MS/MS analysis. Peptides (1 μg) were separated and analyzed with a nano-UPLC (EASYnLC1200) coupled to a Q Exactive HFX Orbitrap instrument (Thermo Fisher Scientific, USA) with a nano-electrospray ion source. Peptides were loaded on a reversed phase column (100 μm ID × 15 cm, Reprosil-Pur 120 C18-AQ, 1.9 μm) (Dr. Maisch GmbH, Ammerbuch, Germany), and separated with a 90 min gradient at 300 nl/min flow rate. Mobile phases were H_2_O with 0.1% FA, 2% ACN (phase A) and 80% ACN, 0.1% FA (phase B). The gradient applied was: 2–5% B for 2 min, 5–22% B for 68 min, 22–45% for 16 min, 45–95% for 2 min, 95% for 2 min. Data dependent acquisition (DDA) was performed in profile and positive mode with Orbitrap analyzer at a resolution of 120,000 (@200 *m/z*) and m/z range of 350–1600 for MS1; for MS2, the resolution was set to 15 k with a fixed first mass of 110 *m/z*. The automatic gain control (AGC) target for MS1 was set to 3E6 with max IT 30 ms, and 1E5 for MS2 with max IT 96 ms. The top 20 most intense ions were fragmented by HCD with normalized collision energy (NCE) of 32%, and isolation window of 0.7 *m/z*. The dynamic exclusion time window was 45 s, single charged peaks and peaks with charge exceeding 6 were excluded from the DDA procedure.

Data analysis. Vendor’s raw MS files were processed using Proteome Discoverer (PD) software (Version 2.4.0.305) and the built-in Sequest HT search engine. MS spectra lists were searched against their species-level UniProt FASTA databases (uniprot-Human-9606–2020-10. fasta), with Carbamidomethyl [C], TMT 6 plex (K), and TMT 6 plex (N-term) as a fixed modification and Oxidation (M) and Acetyl (Protein N-term) as variable modifications. Trypsin was used as protease. The peptide tolerance was set to 10 ppm and MS/MS tolerance was 0.02 Da. A maximum of 2 missed cleavage (s) was allowed. The false discovery rate (FDR) was set to 0.01 for both PSM and peptide levels. Peptide identification was performed with an initial precursor mass deviation of up to 10 ppm and a fragment mass deviation of 0.02 Da. Unique peptide and Razor peptide were used for protein quantification and total peptide amount for normalization. All the other parameters were reserved as default. Student’s t-test was used to identify DEPs between two groups, and p < 0.05 was considered significant.

### Western blot analysis

Total cellular protein in PANC-1 and ASPC-1 cells after treatment was isolated by the addition of 1% PMSF and RIPA lysis buffer. The samples were subjected to for sodium dodecylsulfate-polyacrylamide gel electrophoresis. Then the proteins were transferred onto a PVDF membrane (Millipore, USA). After being blocked for 1 h at room temperature, the membrane was incubated with antibodies to AKT (1:2000, 2920, CST), p-AKT (1:2000, 4060, CST), JNK (1:1000, 9258, CST), p-JNK (1:1000, 4668, CST), cleaved caspase3/caspase3 (1:1000, 9662, CST), caspase12 (1:1000, 55238-1-AP, Proteintech), TGF-β2 (1:1000, BA0526-2, Boster), SMAD2 (1:1000, 5339, CST), p-SMAD2 (1:1000, 18338, CST), and GAPDH (1:8000, 60004-1-lg, Proteintech) overnight. Before detection with an ECL chemiluminescence detection kit (Advansta, USA), proteins were incubated with the corresponding secondary antibodies for 1 h at room temperature. The bands were obtained by GeneGnome 5 (Synoptics Ltd., UK).

### Statistical analysis

Descriptive variables are presented as means ± SD and compared using the t-test. Estimations are presented with 95% confidence intervals. Statistical analysis was performed using SPSS for windows version 19 (IBM, Chicago, IL, USA).

## Supplementary Information

Supplementary Figure S1.

Supplementary Figure S2.

Supplementary Figure S3.

Supplementary Figure S4.

Supplementary Figure S5.

Supplementary Figure S6.

Supplementary Figure S7.

Supplementary Figure S8.

Supplementary Legends.

## Data Availability

All data is available in this paper and related supplemental files.
