# Supplementary figures and images for: Rhoifolin from Plumula Nelumbinis exhibits anti-cancer effects in pancreatic cancer via AKT/JNK signaling pathways
Source: Sci Rep. 2022 Apr 5;12:5654. doi: 10.1038/s41598-022-09581-3 (PMC8983741; doi:10.1038/s41598-022-09581-3)

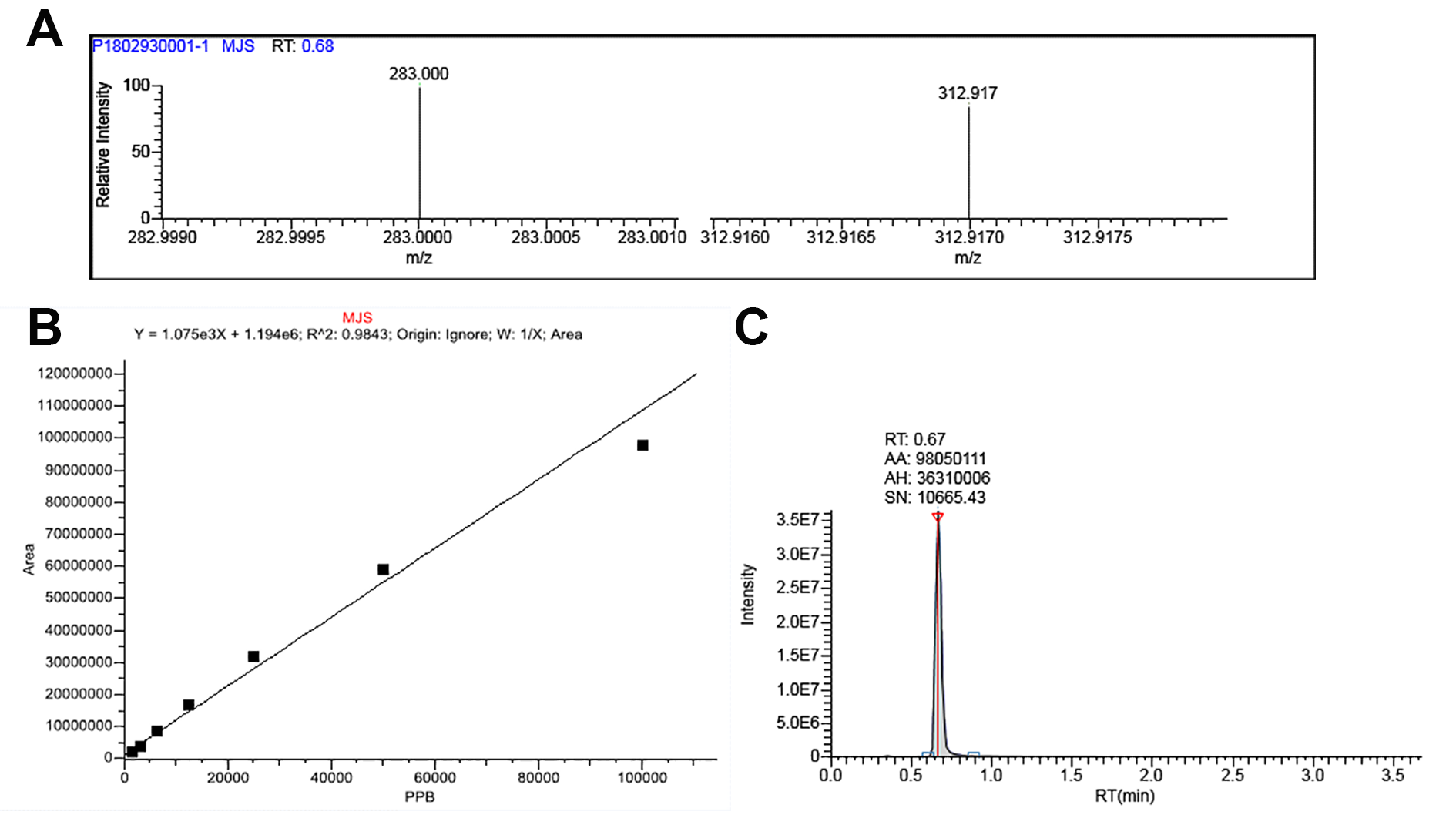

Supplement: Supplementary file 1 — Supplementary Figure S1. [file 41598_2022_9581_MOESM1_ESM.png]

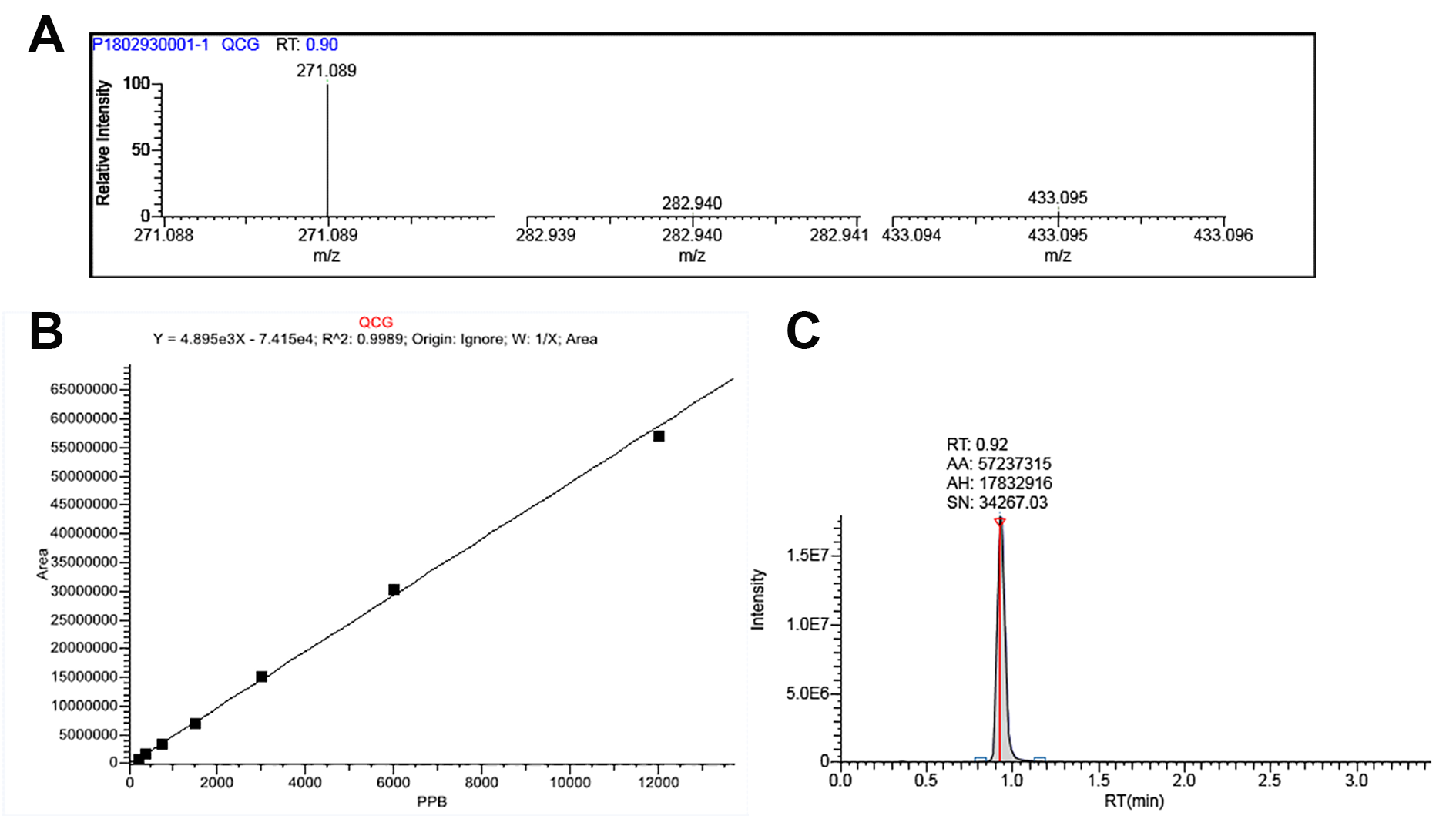

Supplement: Supplementary file 2 — Supplementary Figure S2. [file 41598_2022_9581_MOESM2_ESM.png]

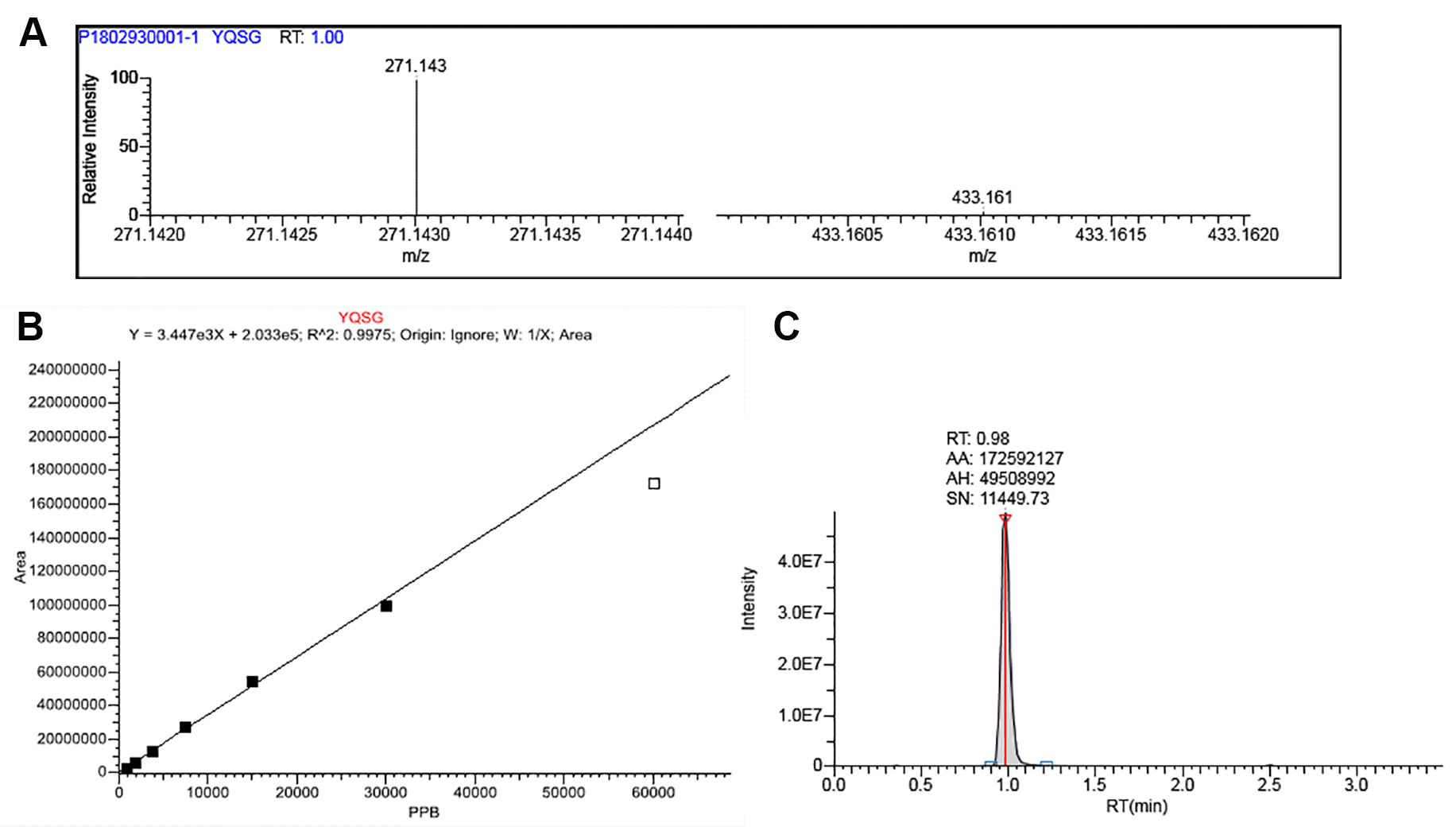

Supplement: Supplementary file 3 — Supplementary Figure S3. [file 41598_2022_9581_MOESM3_ESM.png]

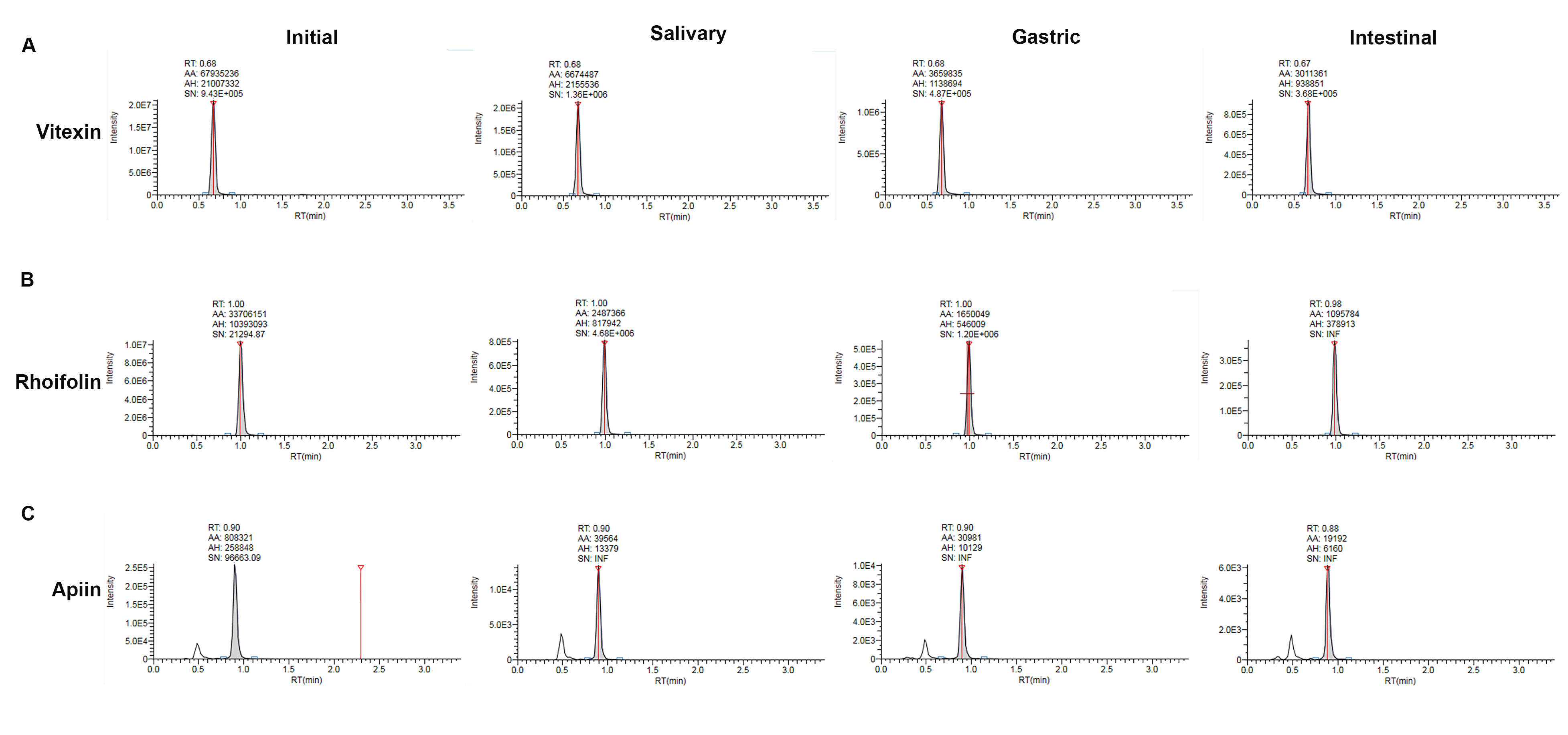

Supplement: Supplementary file 4 — Supplementary Figure S4. [file 41598_2022_9581_MOESM4_ESM.png]

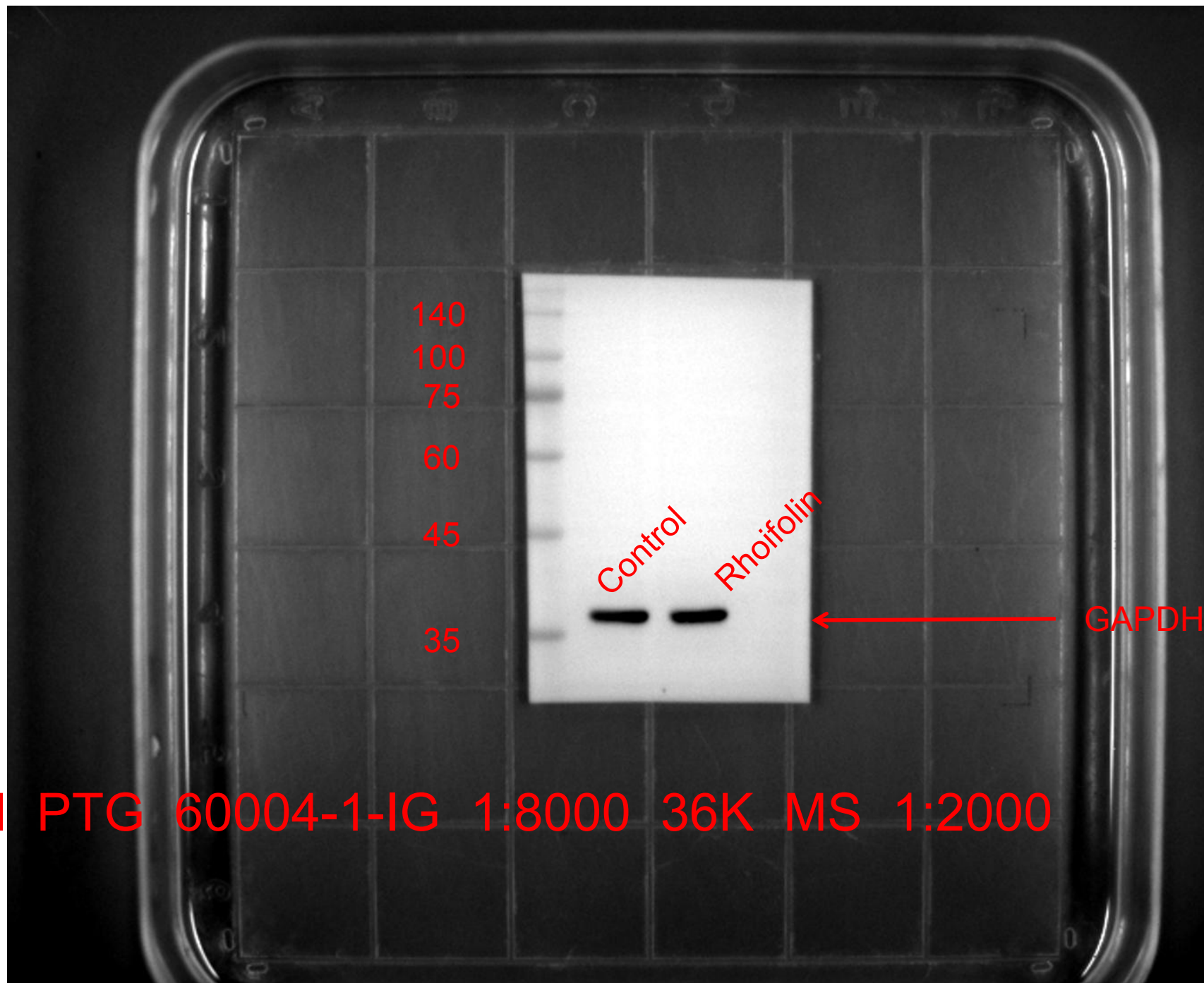

GAPDH PTG 60004-1-IG 1:8000 36K MS 1:2000

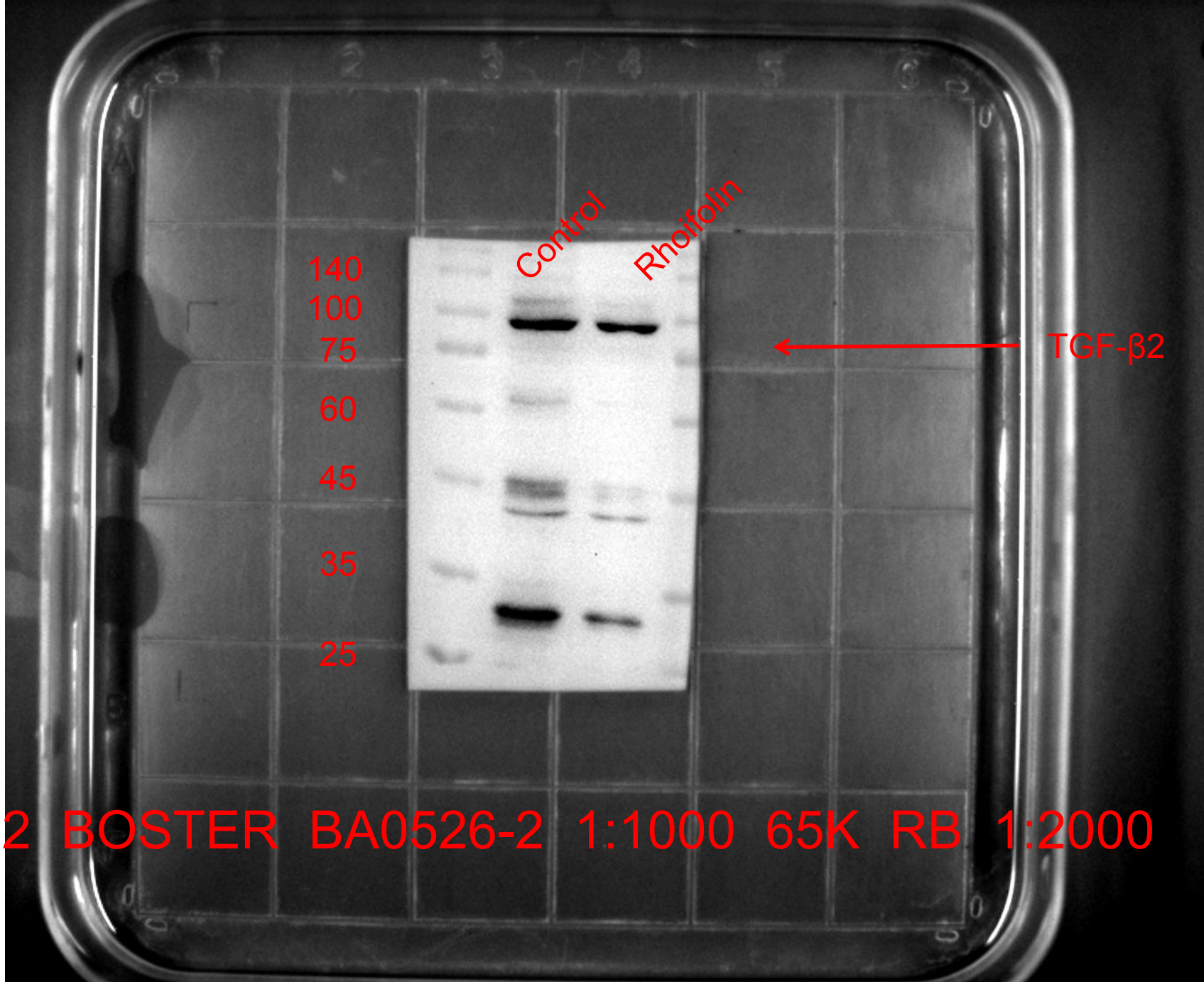

TGF-β2 BOSTER BA0526-2 1:1000 65K RB 1:2000

140  
100  
75  
60  
45  
35  
25

Control

Rhoifolin

← SMAD2

SMAD2 CST 5339 1:1000 60K RB 1:2000

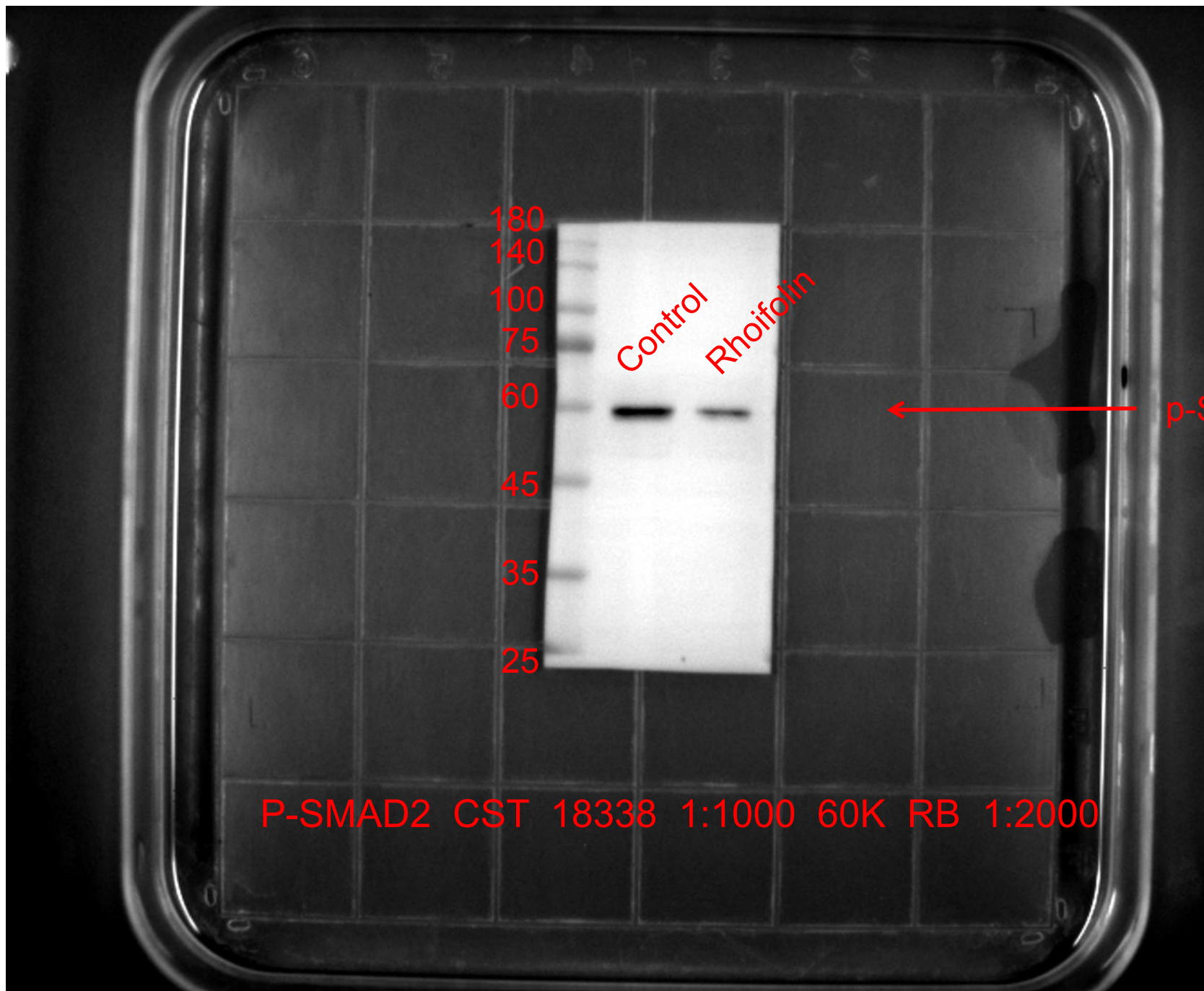

P-SMAD2 CST 18338 1:1000 60K RB 1:2000

Supplement: Supplementary file 8 — Supplementary Figure S8. [file 41598_2022_9581_MOESM8_ESM.pdf]
