# Supplementary material for: Rhoifolin from Plumula Nelumbinis exhibits anti-cancer effects in pancreatic cancer via AKT/JNK signaling pathways
Source: Sci Rep. 2022 Apr 5;12:5654. doi: 10.1038/s41598-022-09581-3 (PMC8983741; doi:10.1038/s41598-022-09581-3)
Supplement: Supplementary file 5 — Supplementary Figure S5. [file 41598_2022_9581_MOESM5_ESM.pdf]

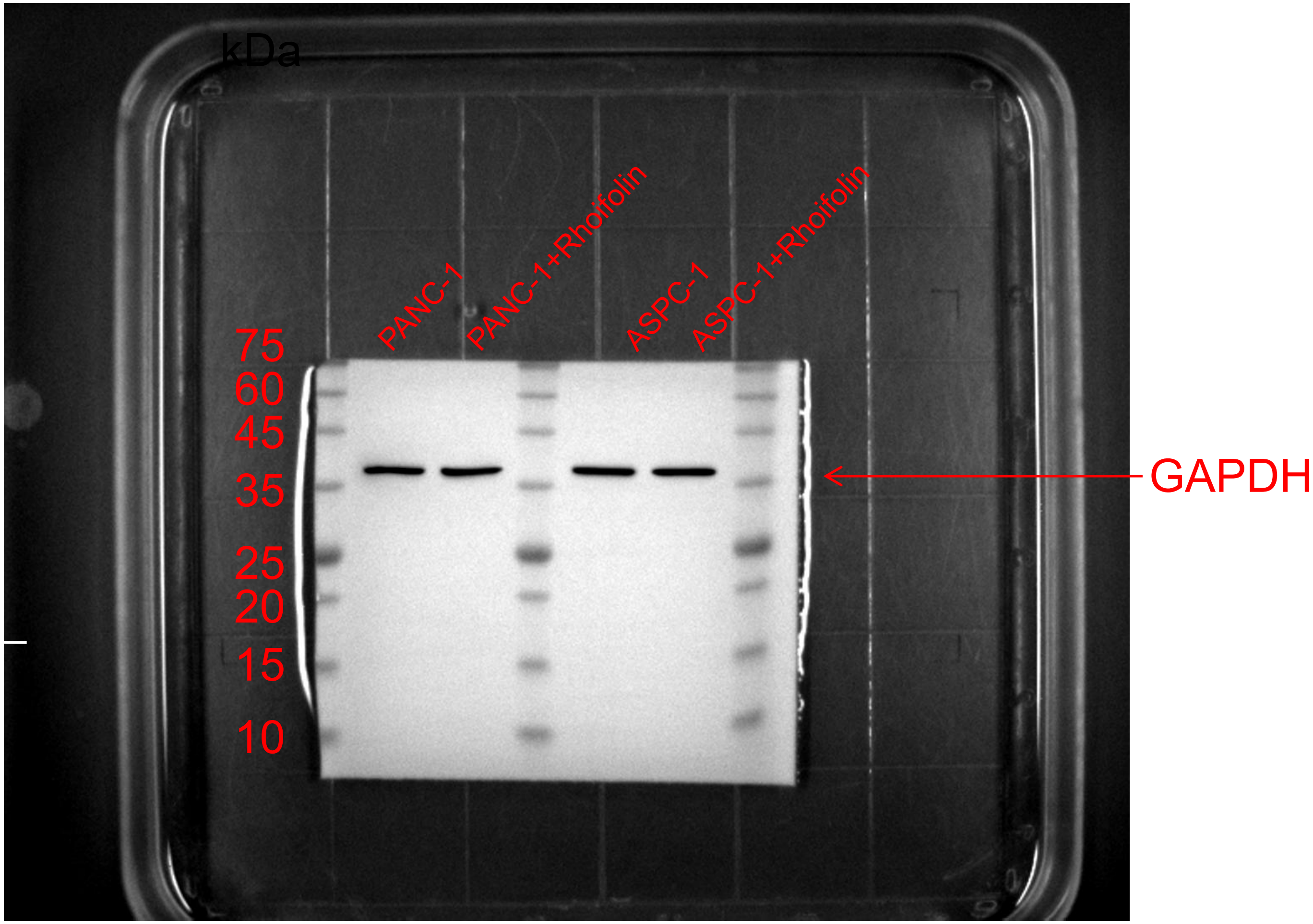

GAPDH, Proteintech, 60004-1-Ig, 1:8000, 36kD; anti-Mouse IgG, Jackson, 115-035-003, 1:5000

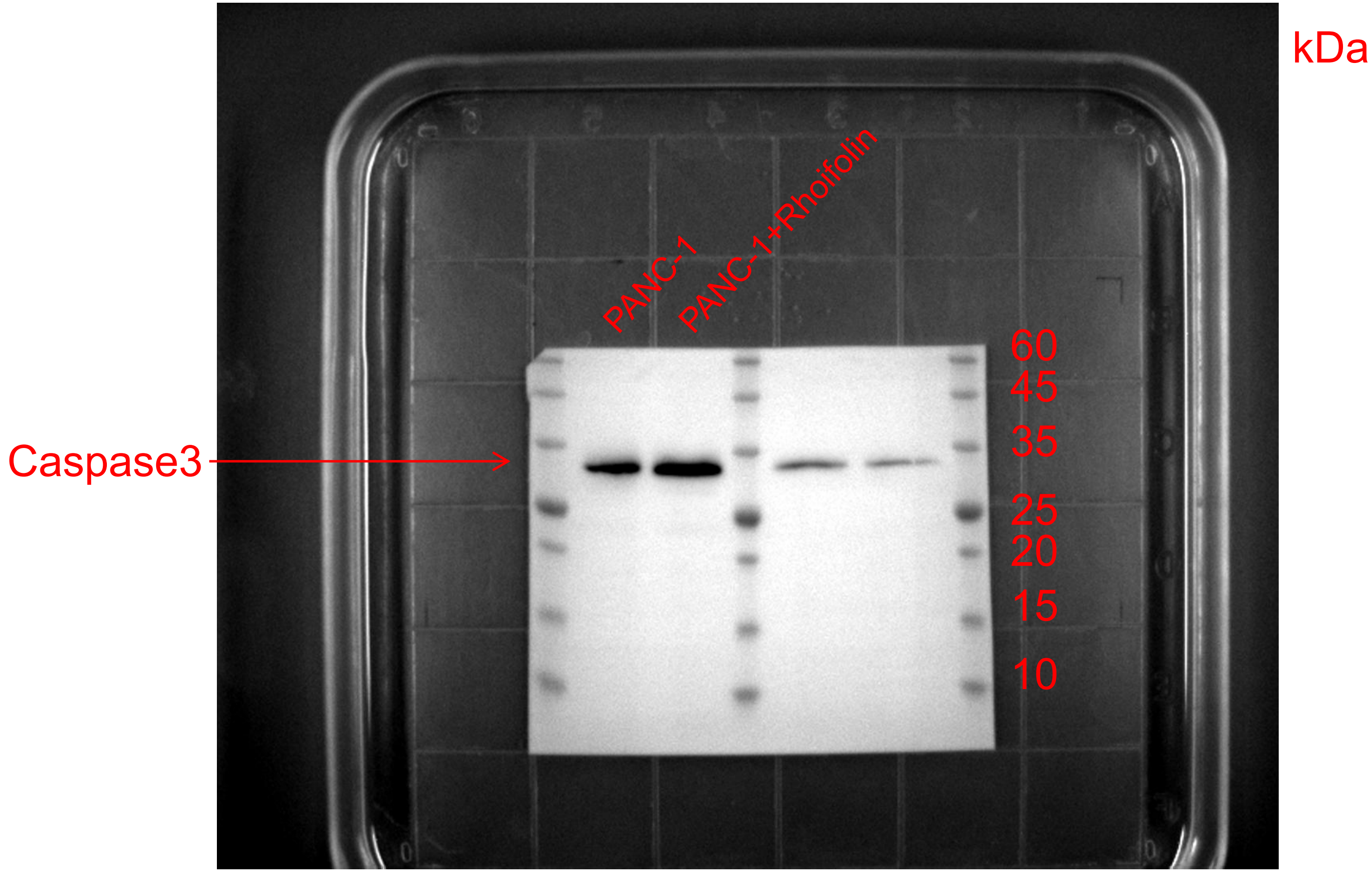

Caspase3, CST, 9662, 1:1000, 17/19/35kD; anti-Rabbit IgG, Jackson, 111-035-003, 1:2000

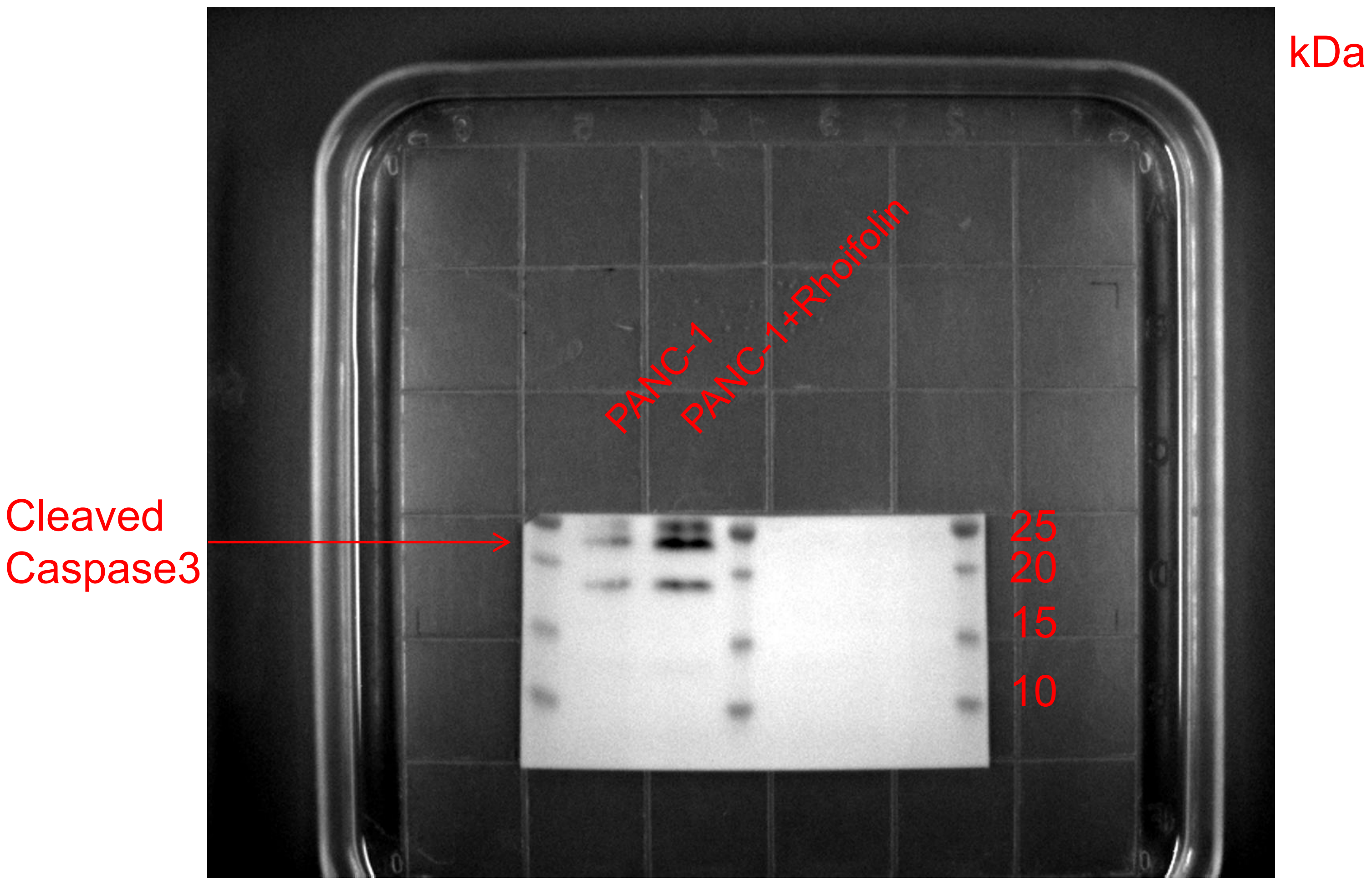

Caspase3, CST, 9662, 1:1000, 17/19/35kD; anti-Rabbit IgG, Jackson, 111-035-003, 1:2000

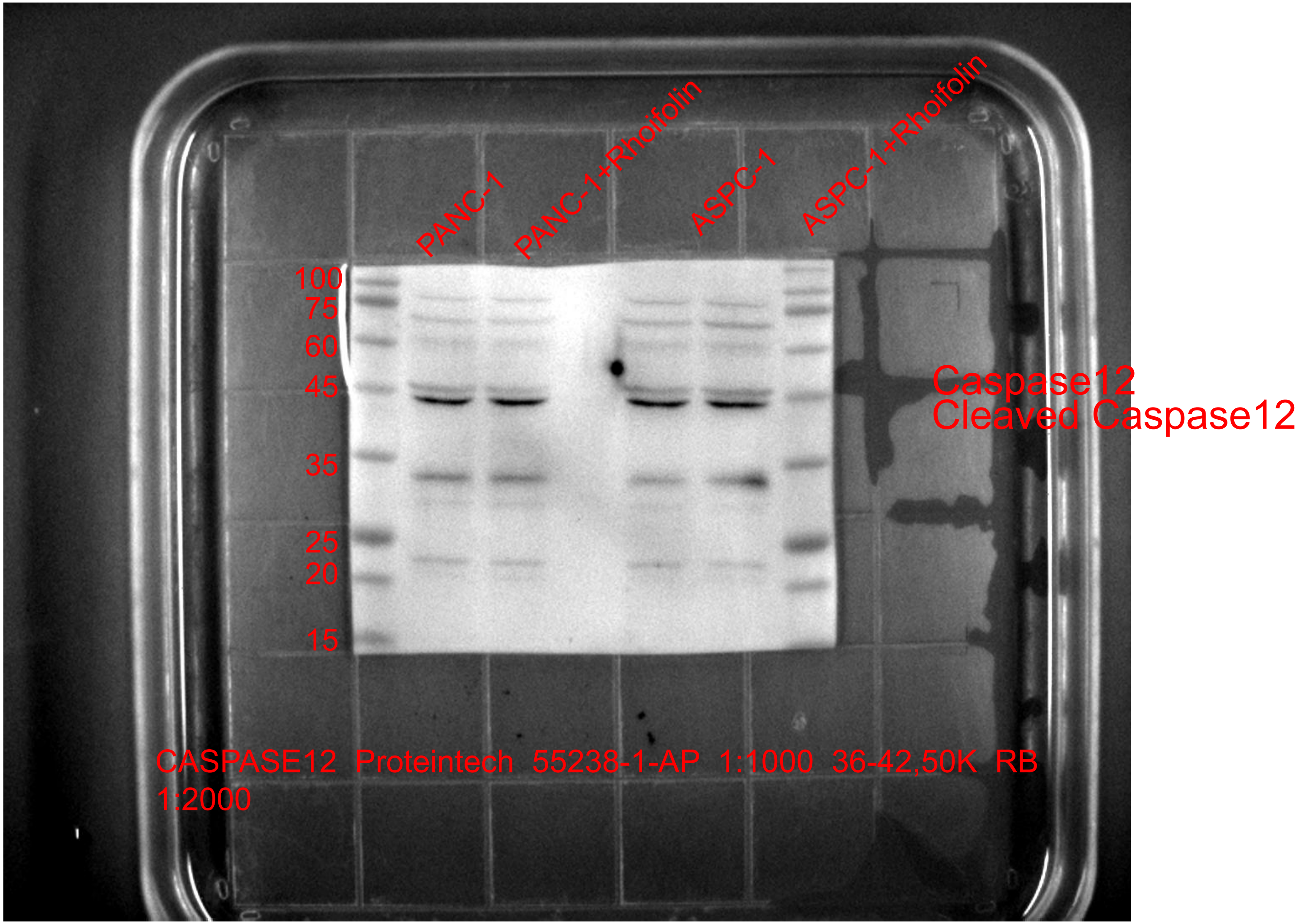
PANC-1
PANC-1+Rhoifolin
ASPC-1
ASPC-1+Rhoifolin
100
75
60
45
35
25
20
15
Caspase12
Cleaved Caspase12
CASPASE12 Proteintech 55238-1-AP 1:1000 36-42,50K RB
1:2000

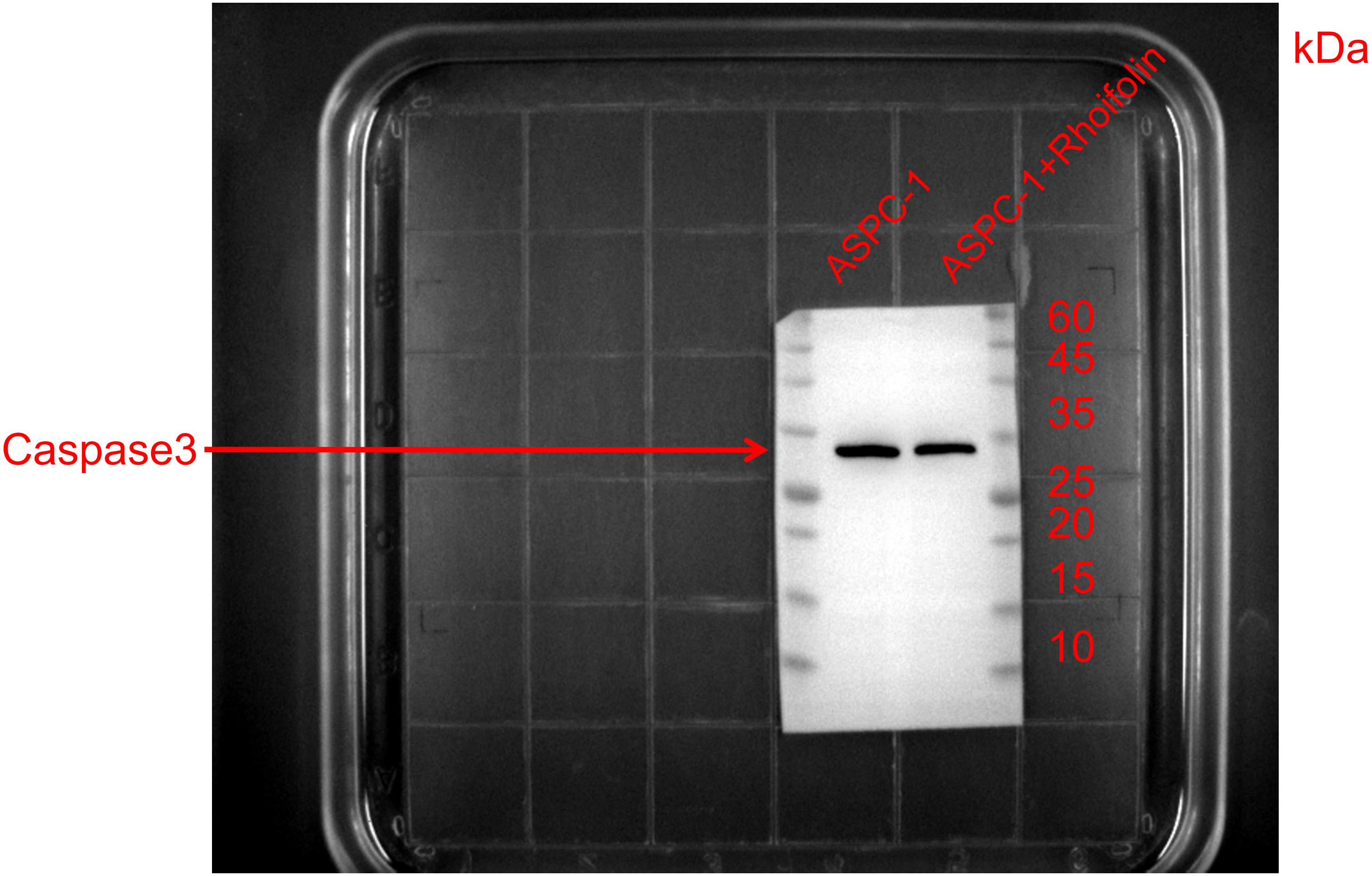

Caspase3, CST, 9662, 1:1000, 17/19/35kD; anti-Rabbit IgG, Jackson, 111-035-003, 1:2000

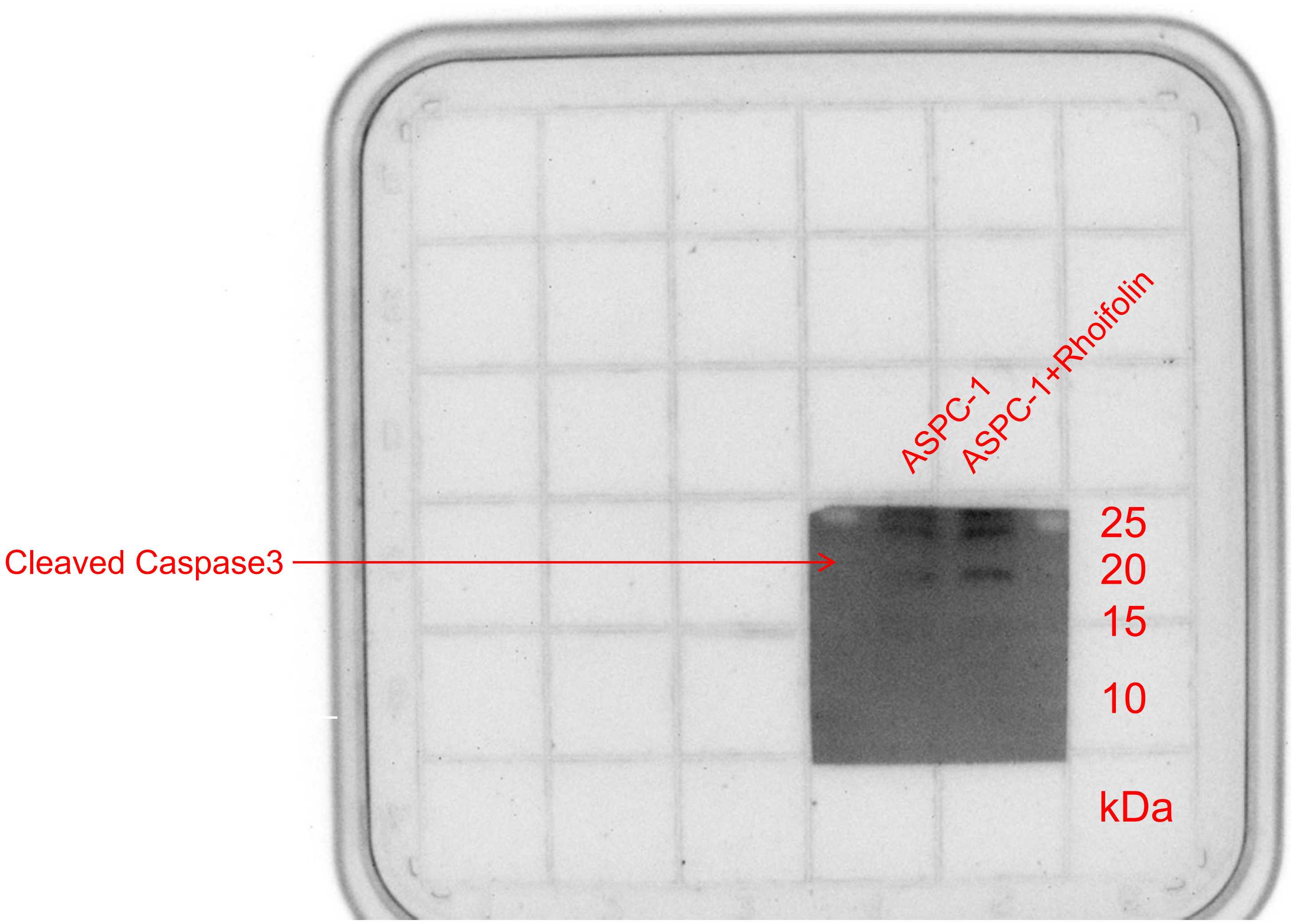

Caspase3, CST, 9662, 1:1000, 17/19/35kD; anti-Rabbit IgG, Jackson, 111-035-003, 1:2000
