# Supplementary material for: Rhoifolin from Plumula Nelumbinis exhibits anti-cancer effects in pancreatic cancer via AKT/JNK signaling pathways
Source: Sci Rep. 2022 Apr 5;12:5654. doi: 10.1038/s41598-022-09581-3 (PMC8983741; doi:10.1038/s41598-022-09581-3)
Supplement: Supplementary file 6 — Supplementary Figure S6. [file 41598_2022_9581_MOESM6_ESM.pdf]

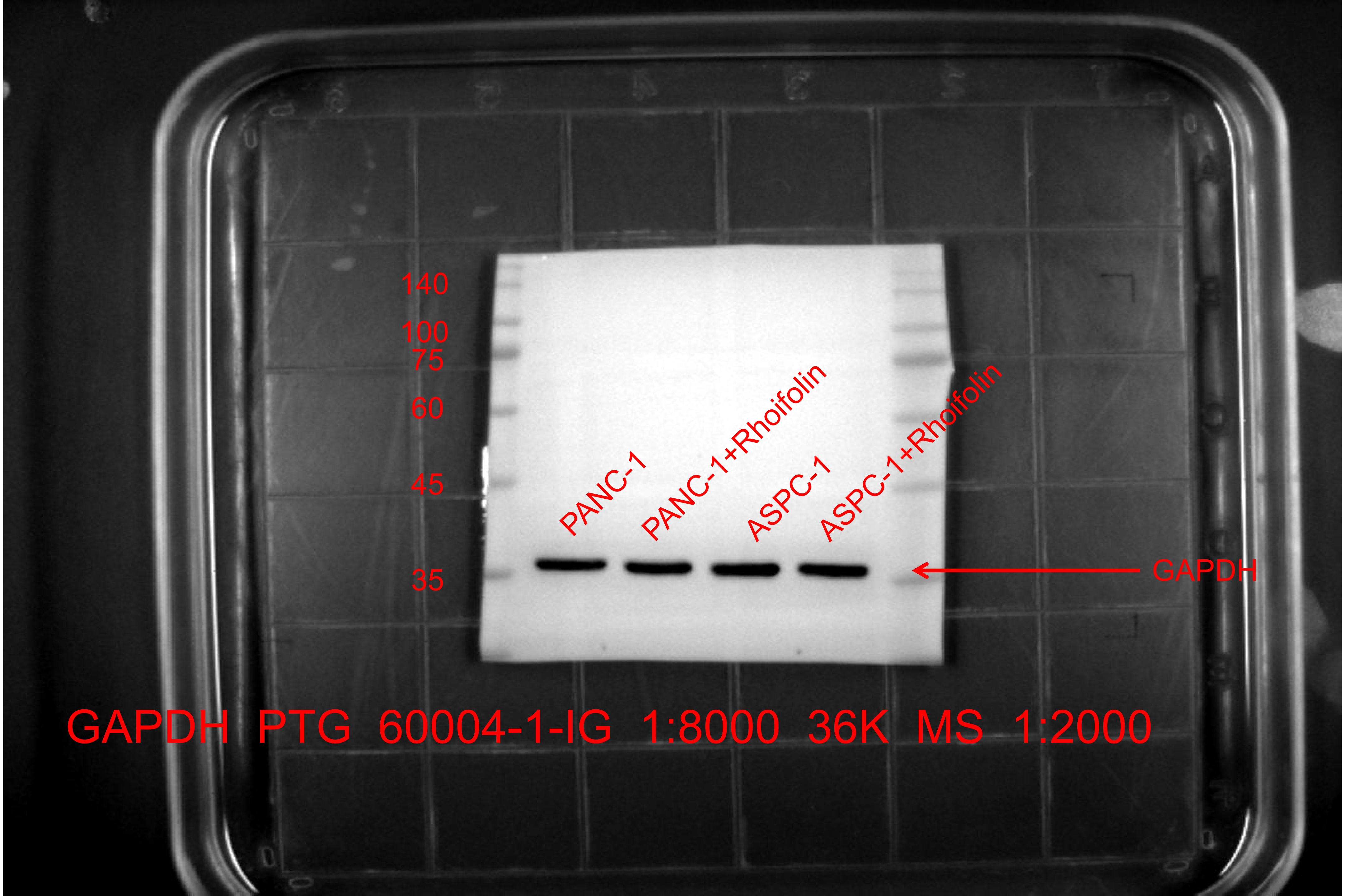
140
100
75
60
45
35
PANC-1
PANC-1+Rhoifolin
ASPC-1
ASPC-1+Rhoifolin
GAPDH
GAPDH PTG 60004-1-IG 1:8000 36K MS 1:2000

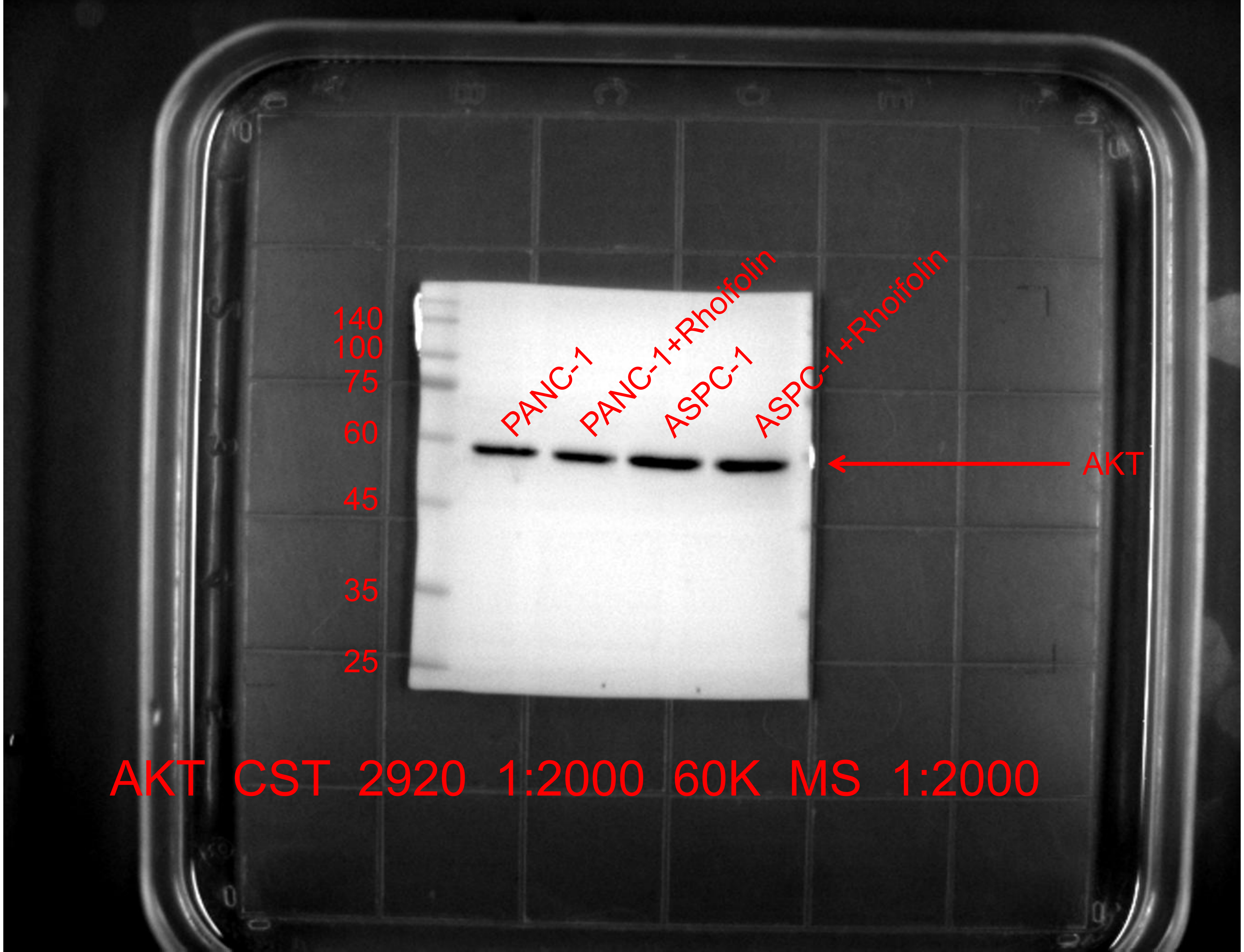
140
100
75
60
45
35
25
PANC-1
PANC-1+Rhoifolin
ASPC-1
ASPC-1+Rhoifolin
AKT
AKT CST 2920 1:2000 60K MS 1:2000

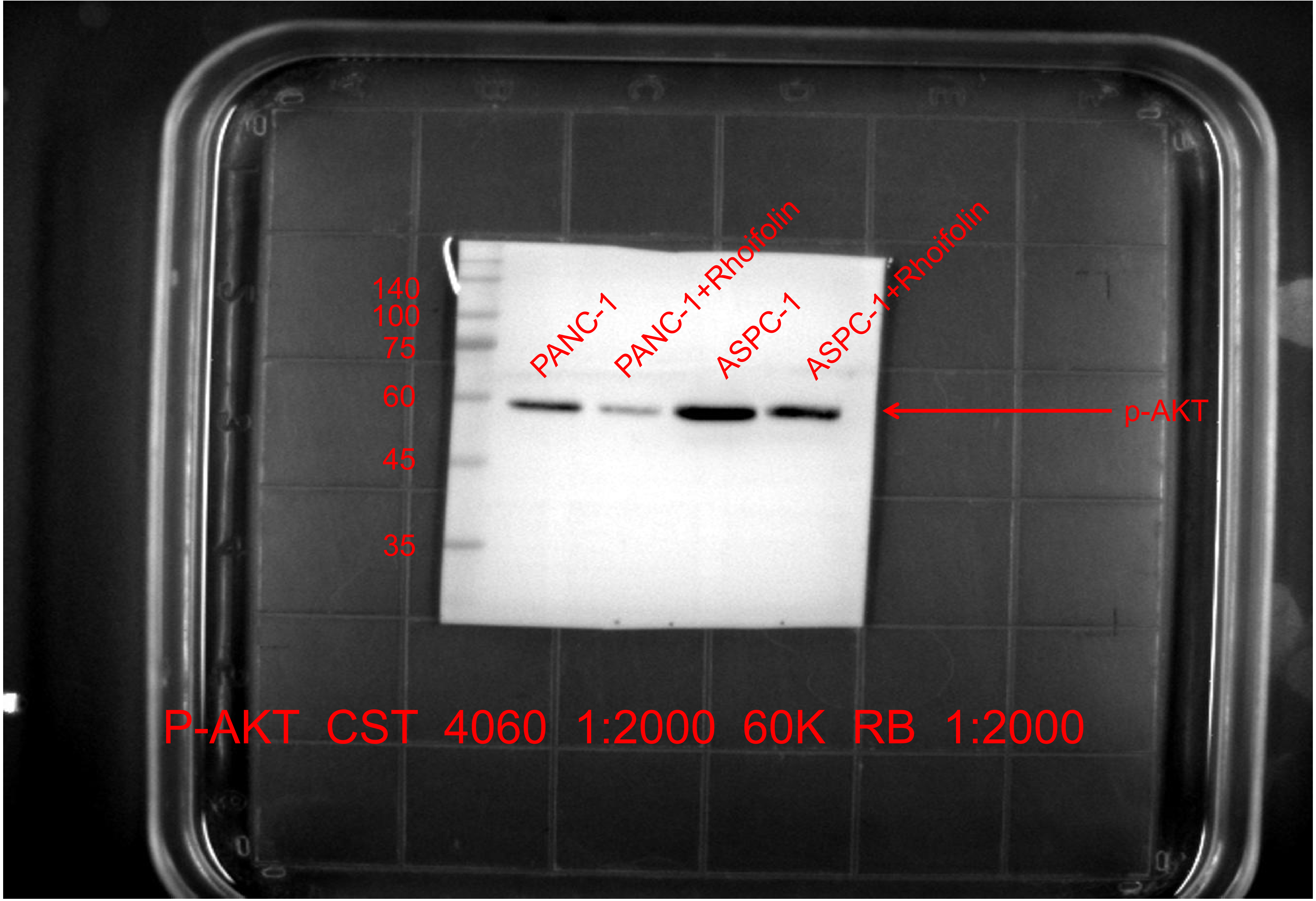
140
100
75
60
45
35
PANC-1
PANC-1+Rhoifolin
ASPC-1
ASPC-1+Rhoifolin
p-AKT
P-AKT CST 4060 1:2000 60K RB 1:2000

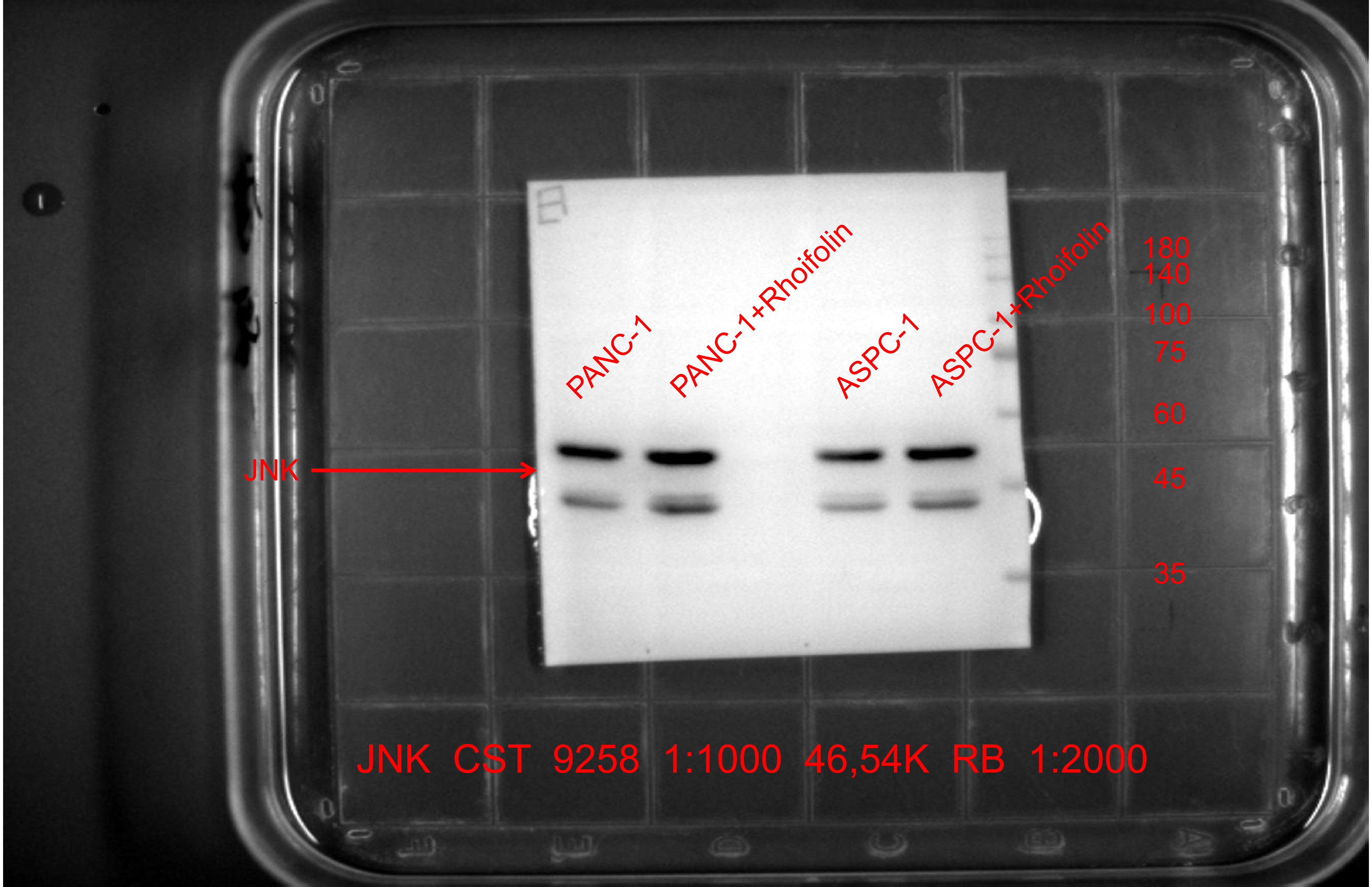
PANC-1
PANC-1+Rhoifolin
ASPC-1
ASPC-1+Rhoifolin
180
140
100
75
60
45
35
JNK
JNK CST 9258 1:1000 46,54K RB 1:2000

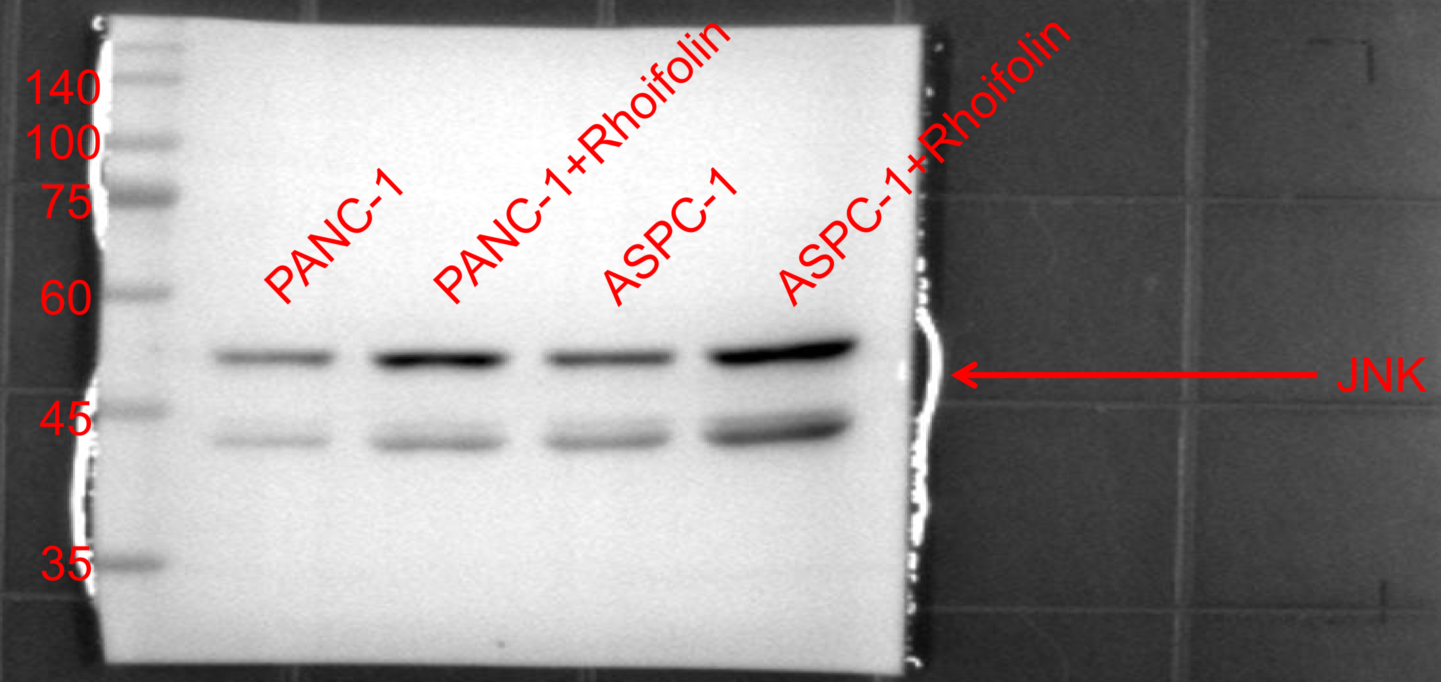

P-JNK, CST, 4668, 1:1000, 46/54kD; anti-Rabbit IgG, Jackson, 111-035-003, 1:2000
