# Supplementary material for: Rhoifolin from Plumula Nelumbinis exhibits anti-cancer effects in pancreatic cancer via AKT/JNK signaling pathways
Source: Sci Rep. 2022 Apr 5;12:5654. doi: 10.1038/s41598-022-09581-3 (PMC8983741; doi:10.1038/s41598-022-09581-3)
Supplement: Supplementary file 7 — Supplementary Figure S7. [file 41598_2022_9581_MOESM7_ESM.pdf]

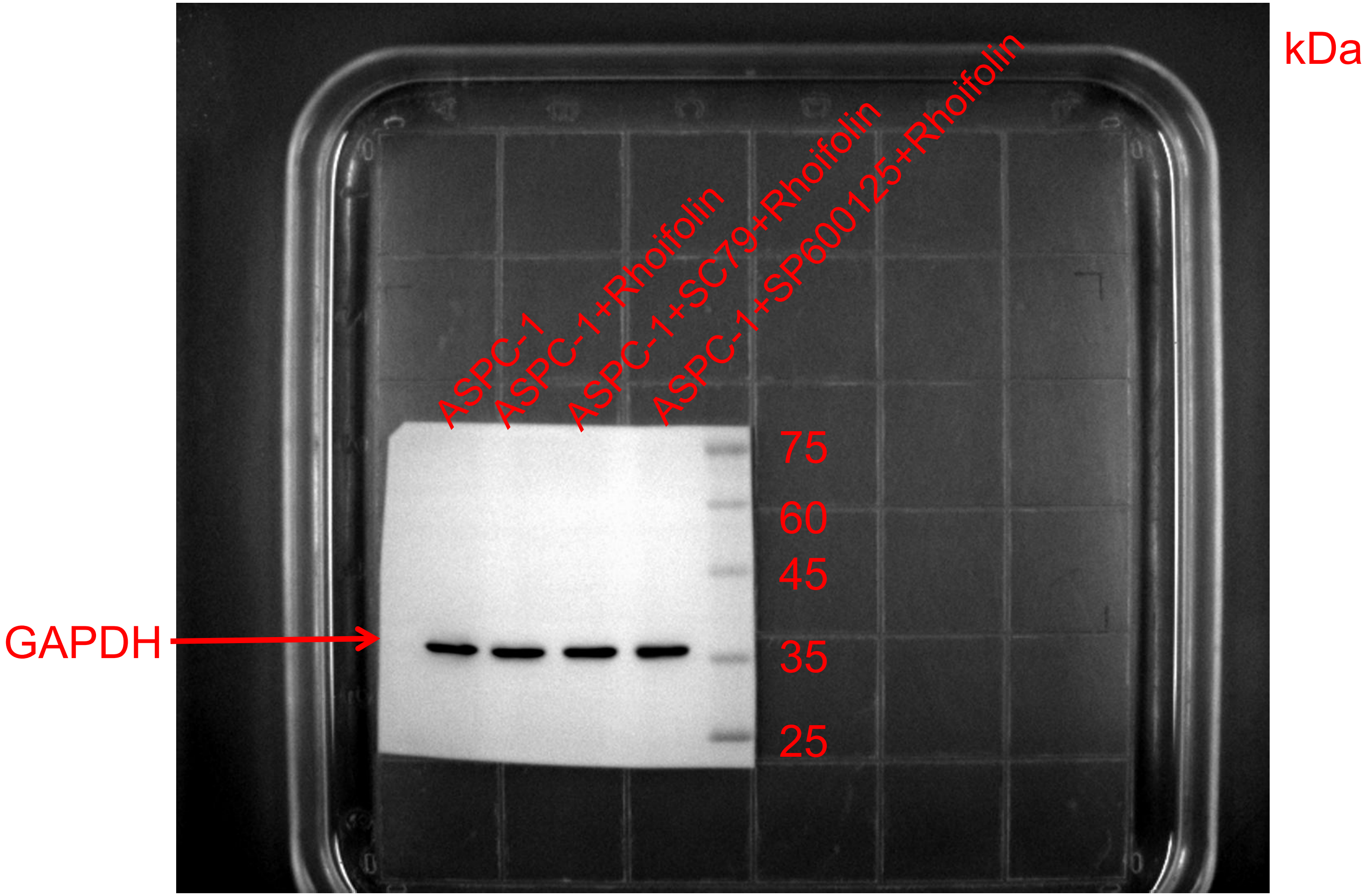

GAPDH, Proteintech, 60004-1-lg, 1:8000, 36kD; anti-Mouse IgG, Jackson, 115-035-003, 1:5000

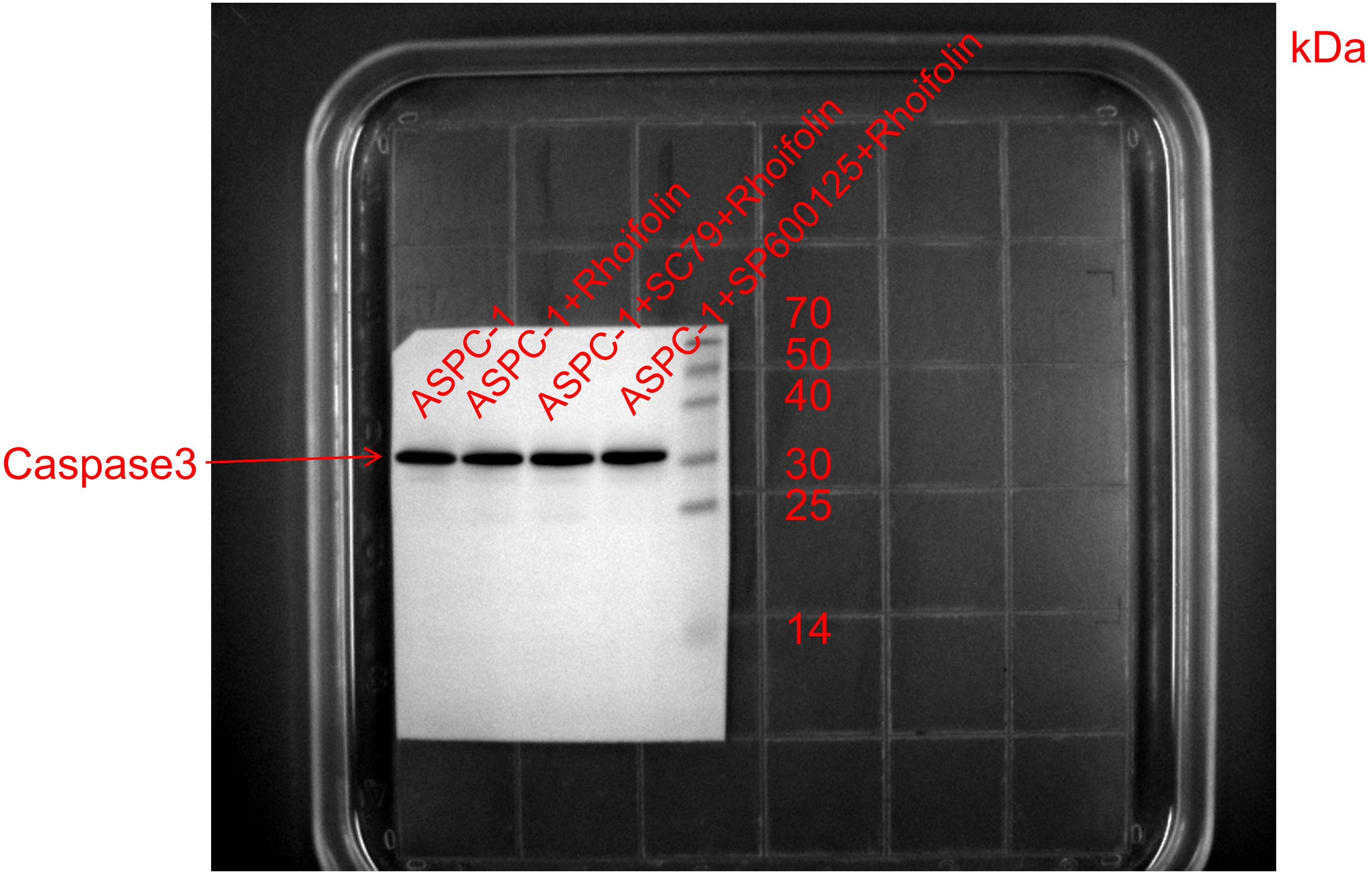

Caspase3, CST, 9662, 1:1000, 17/19/35kD; anti-Rabbit IgG, Jackson, 111-035-003, 1:2000

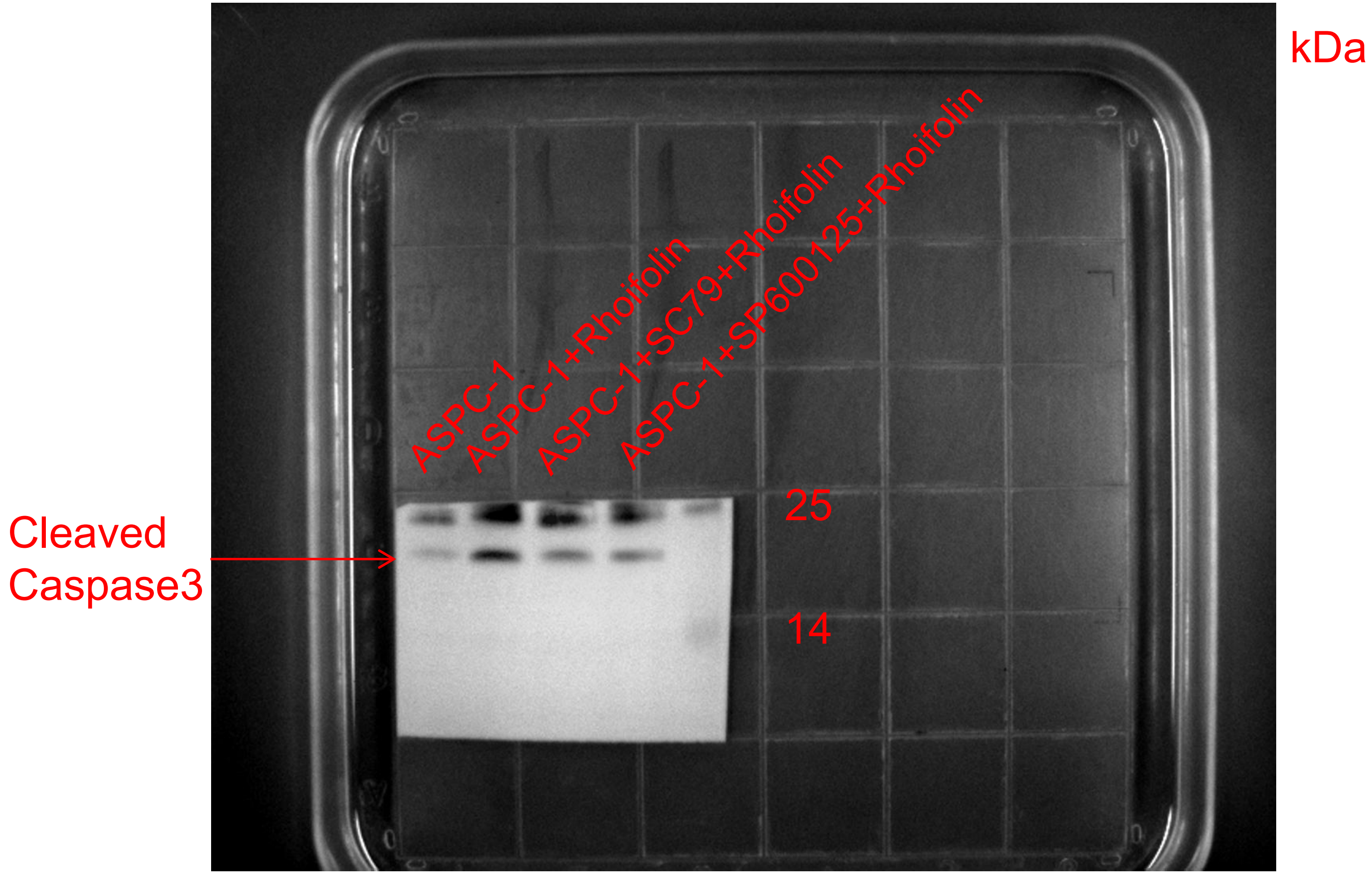

Caspase3, CST, 9662, 1:1000, 17/19/35kD; anti-Rabbit IgG, Jackson, 111-035-003, 1:2000

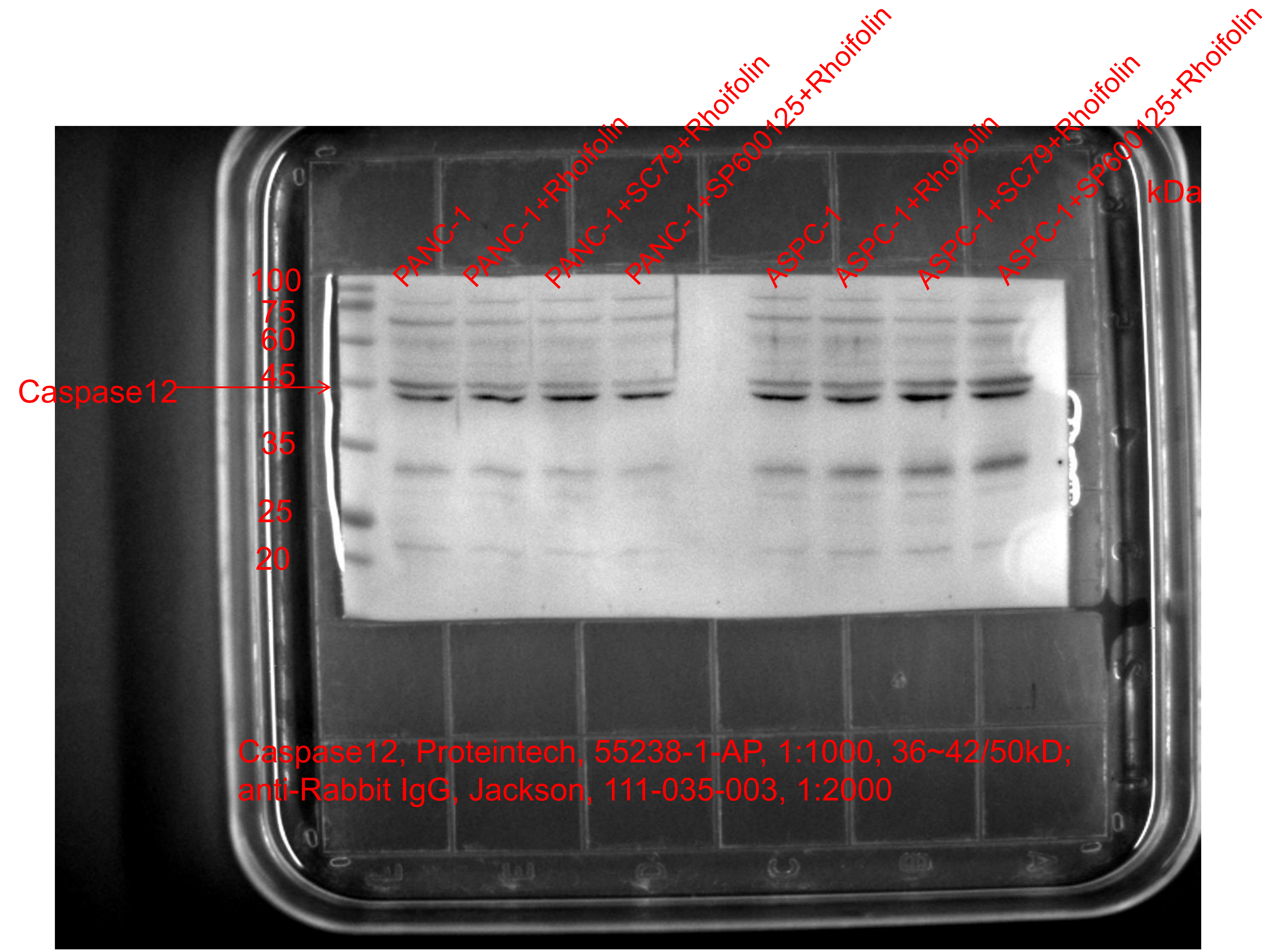
PANC-1
PANC-1+Rhoifolin
PANC-1+SC79+Rhoifolin
PANC-1+SP600125+Rhoifolin
ASPC-1
ASPC-1+Rhoifolin
ASPC-1+SC79+Rhoifolin
ASPC-1+SP600125+Rhoifolin
kDa
100
75
60
45
35
25
20
Caspase12
Caspase12, Proteintech, 55238-1-AP, 1:1000, 36~42/50kD;
anti-Rabbit IgG, Jackson, 111-035-003, 1:2000

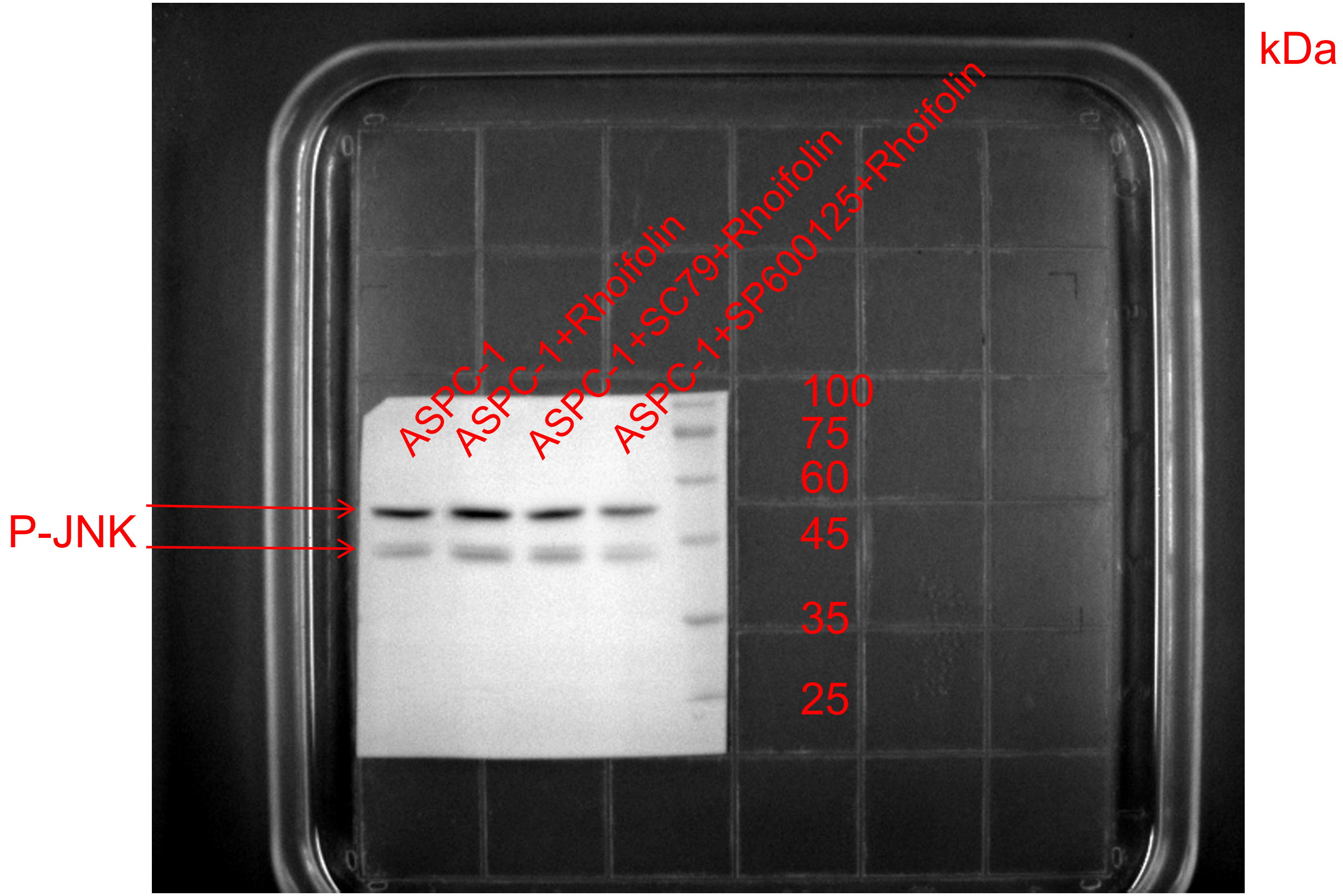

P-JNK, CST, 4668, 1:1000, 46/54kD; anti-Rabbit IgG, Jackson, 111-035-003, 1:2000

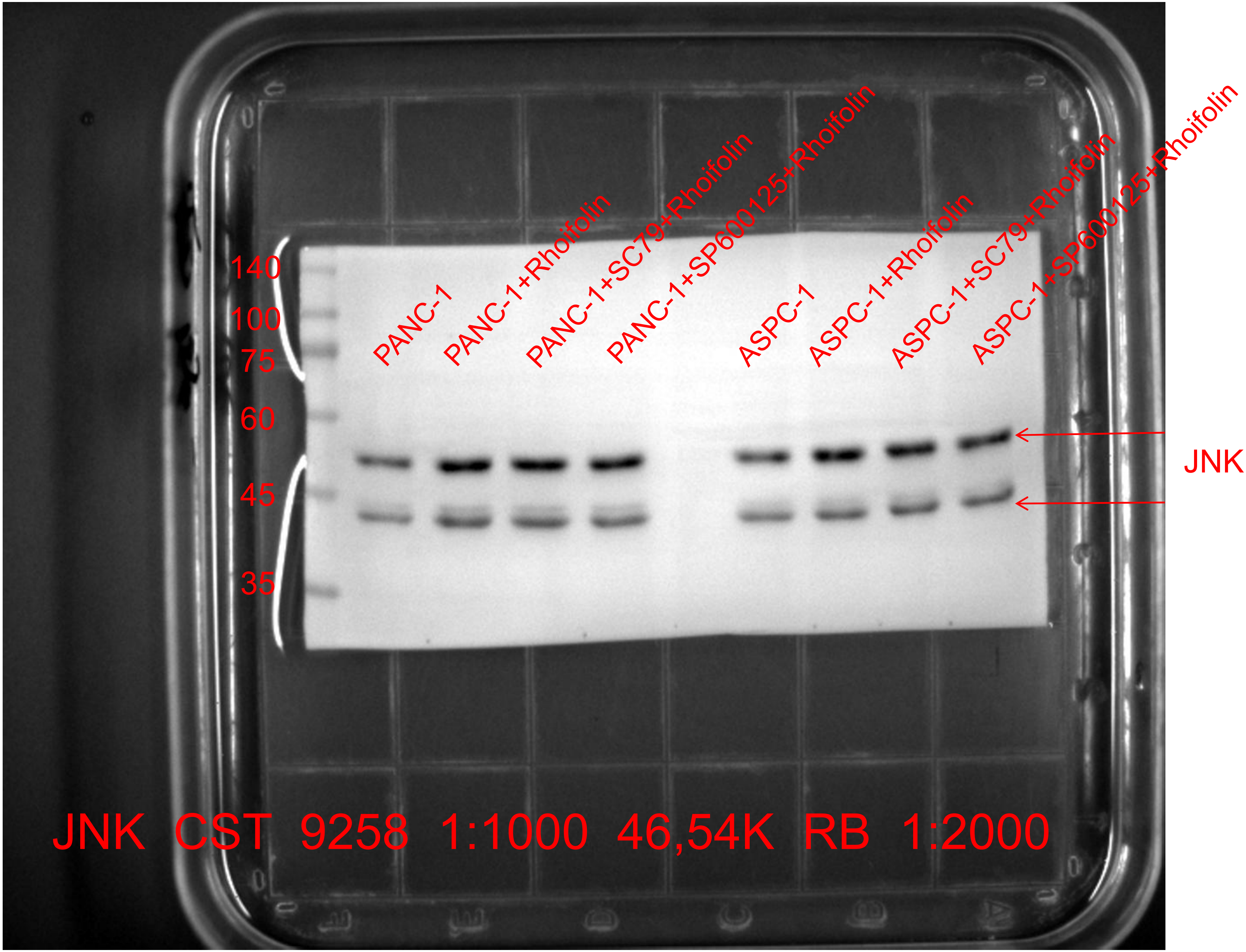
PANC-1
PANC-1+Rhoifolin
PANC-1+SC79+Rhoifolin
PANC-1+SP600125+Rhoifolin
ASPC-1
ASPC-1+Rhoifolin
ASPC-1+SC79+Rhoifolin
ASPC-1+SP600125+Rhoifolin
140
100
75
60
45
35
JNK
JNK CST 9258 1:1000 46,54K RB 1:2000

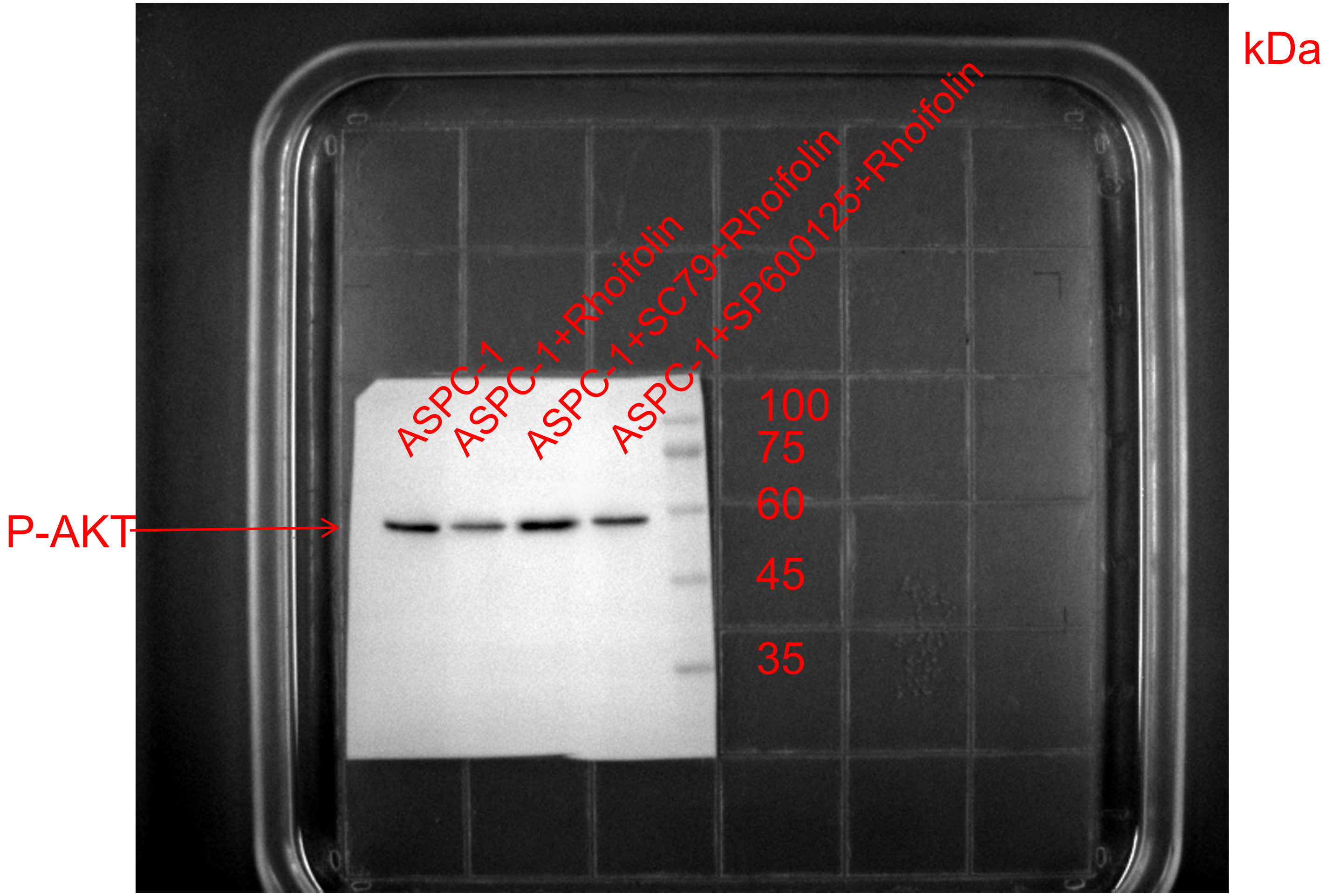

P-AKT, CST, 4060, 1:2000, 60kD;anti-Rabbit IgG, Jackson, 111-035-003, 1:2000

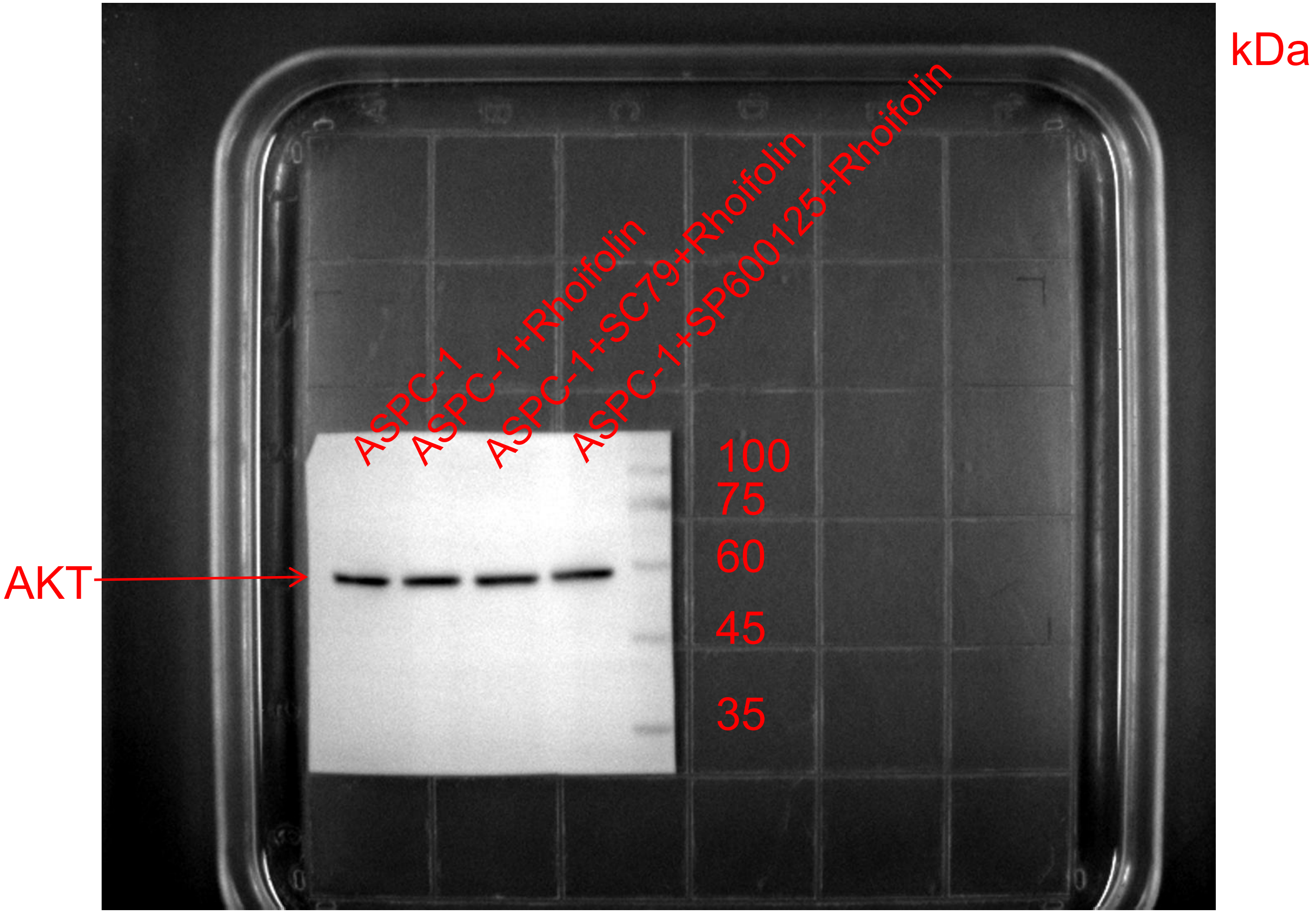

AKT, CST, 2920, 1:2000, 60kD; anti-Mouse IgG, Jackson, 115-035-003, 1:2000

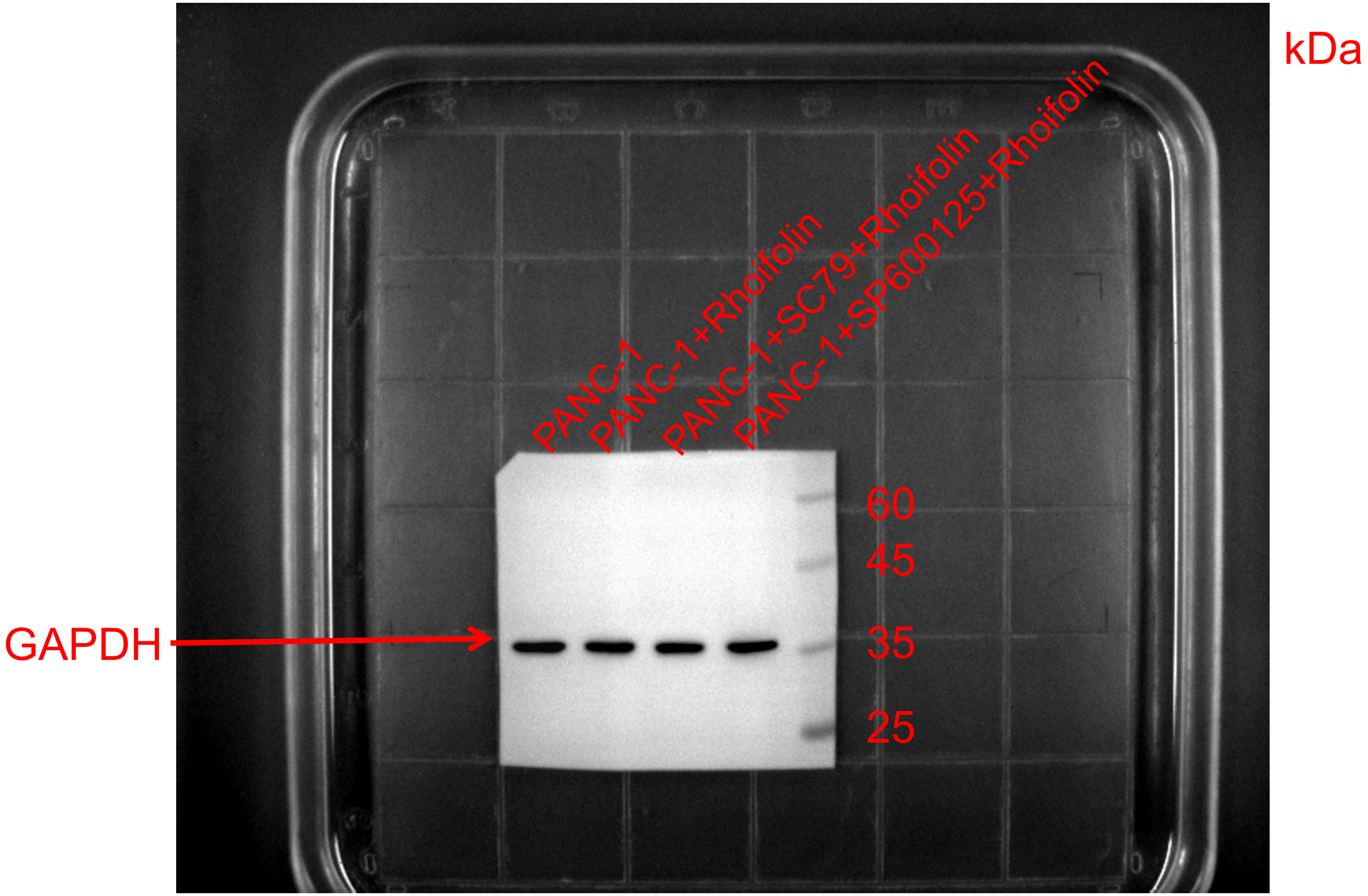

GAPDH, Proteintech, 60004-1-Ig, 1:8000, 36kD; anti-Mouse IgG, Jackson, 115-035-003, 1:5000

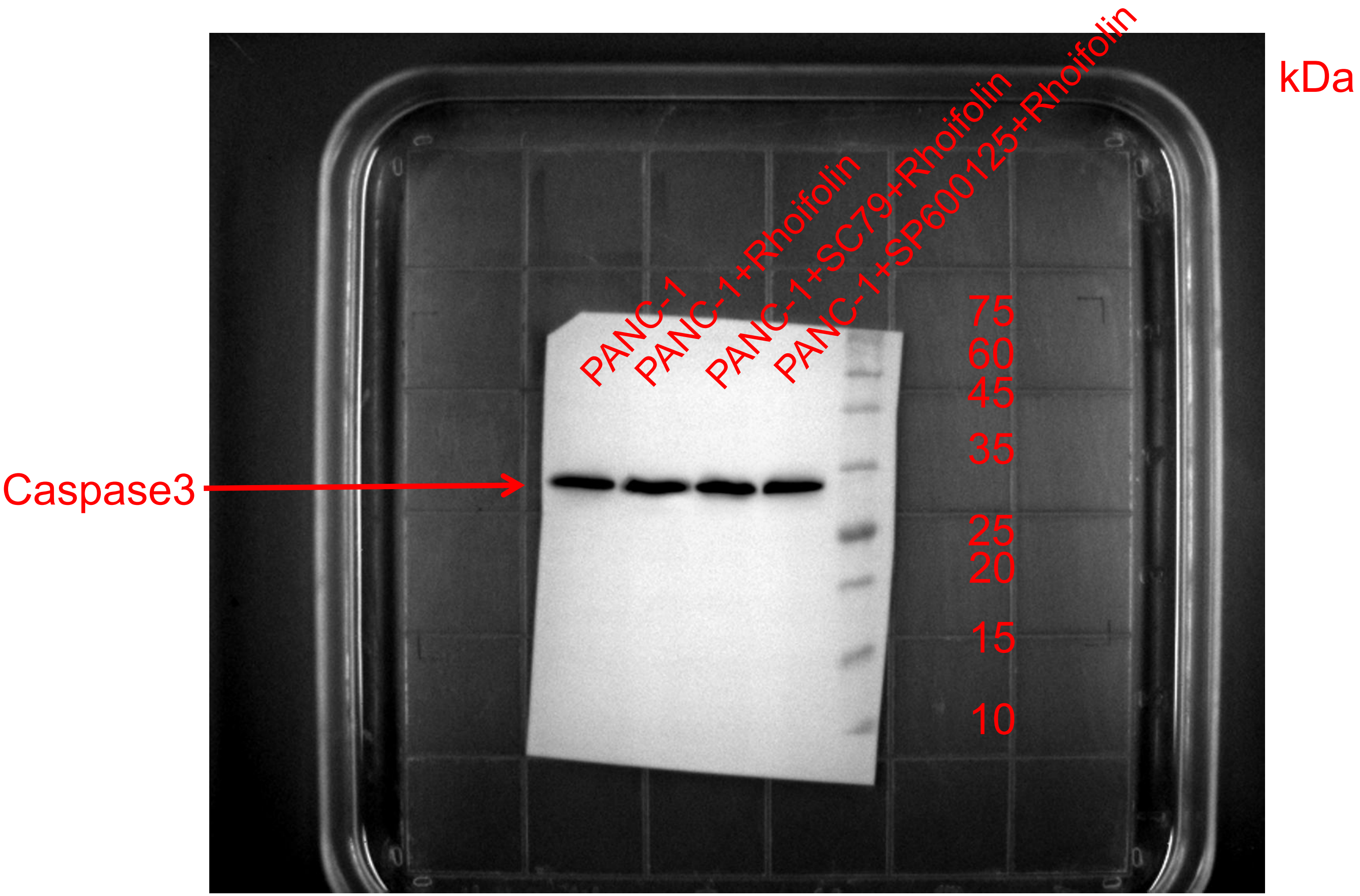

Caspase3, CST, 9662, 1:1000, 17/19/35kD; anti-Rabbit IgG, Jackson, 111-035-003, 1:2000

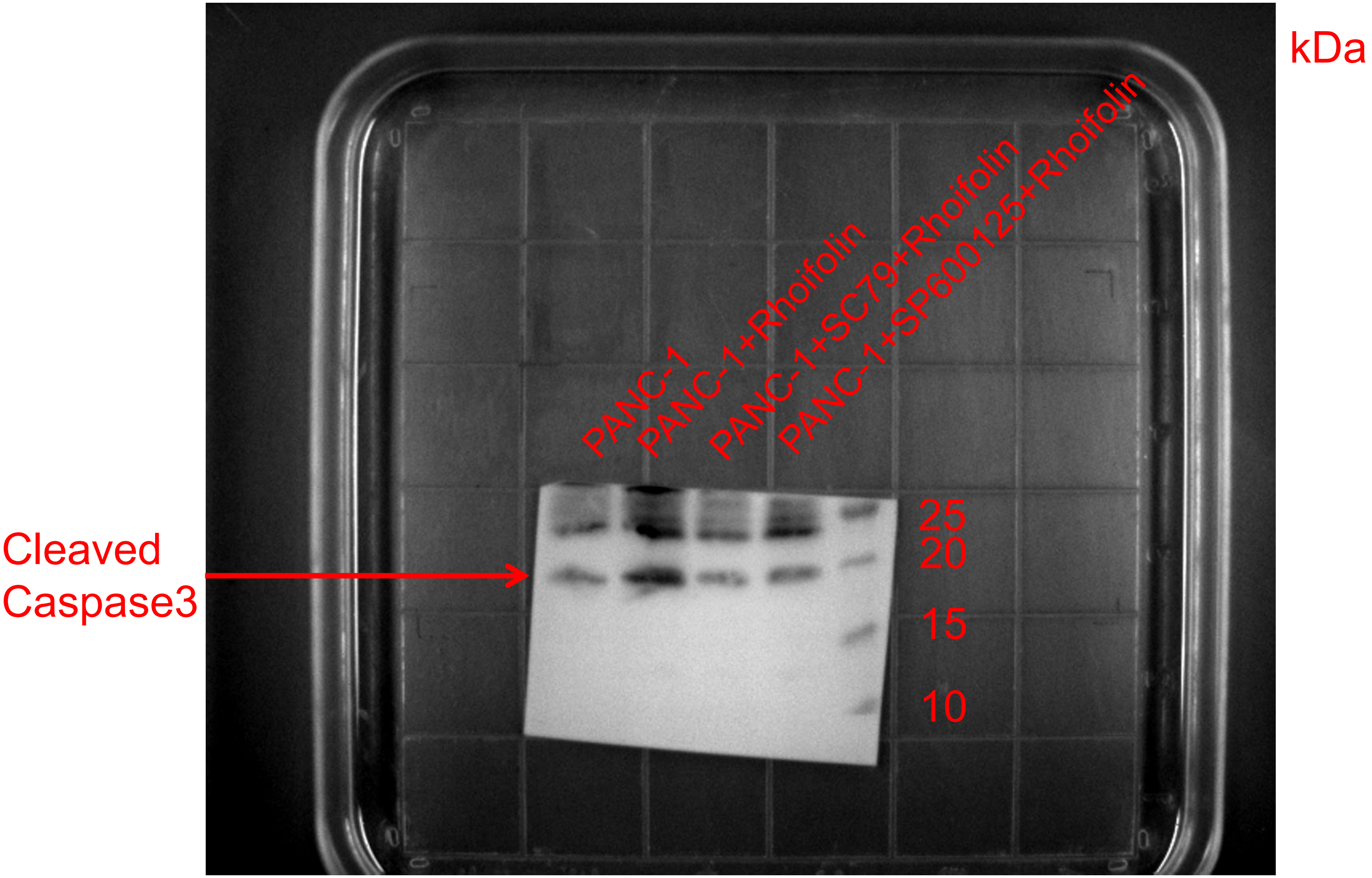

Caspase3, CST, 9662, 1:1000, 17/19/35kD; anti-Rabbit IgG, Jackson, 111-035-003, 1:2000

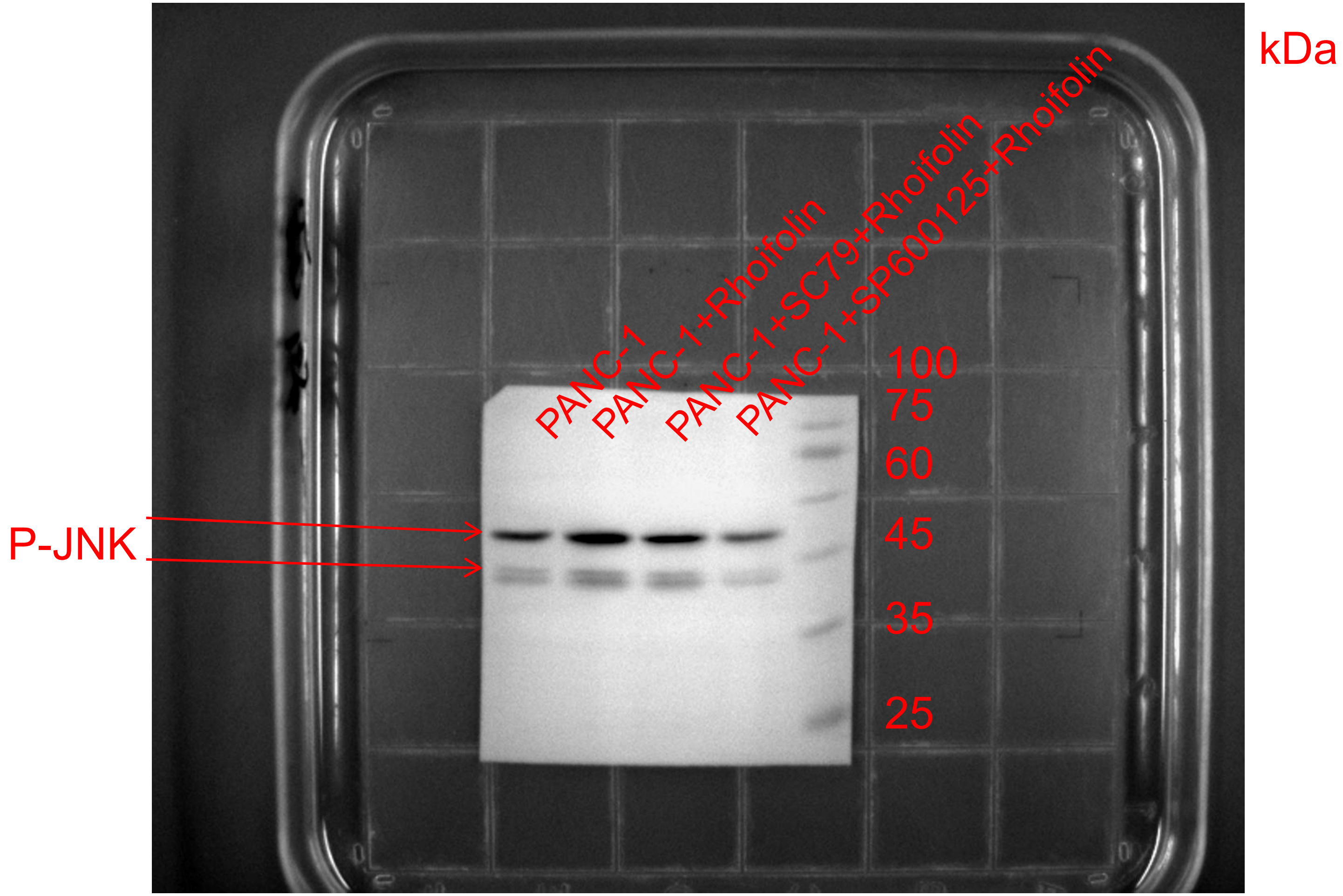

P-JNK, CST, 4668, 1:1000, 46/54kD; anti-Rabbit IgG, Jackson, 111-035-003, 1:2000

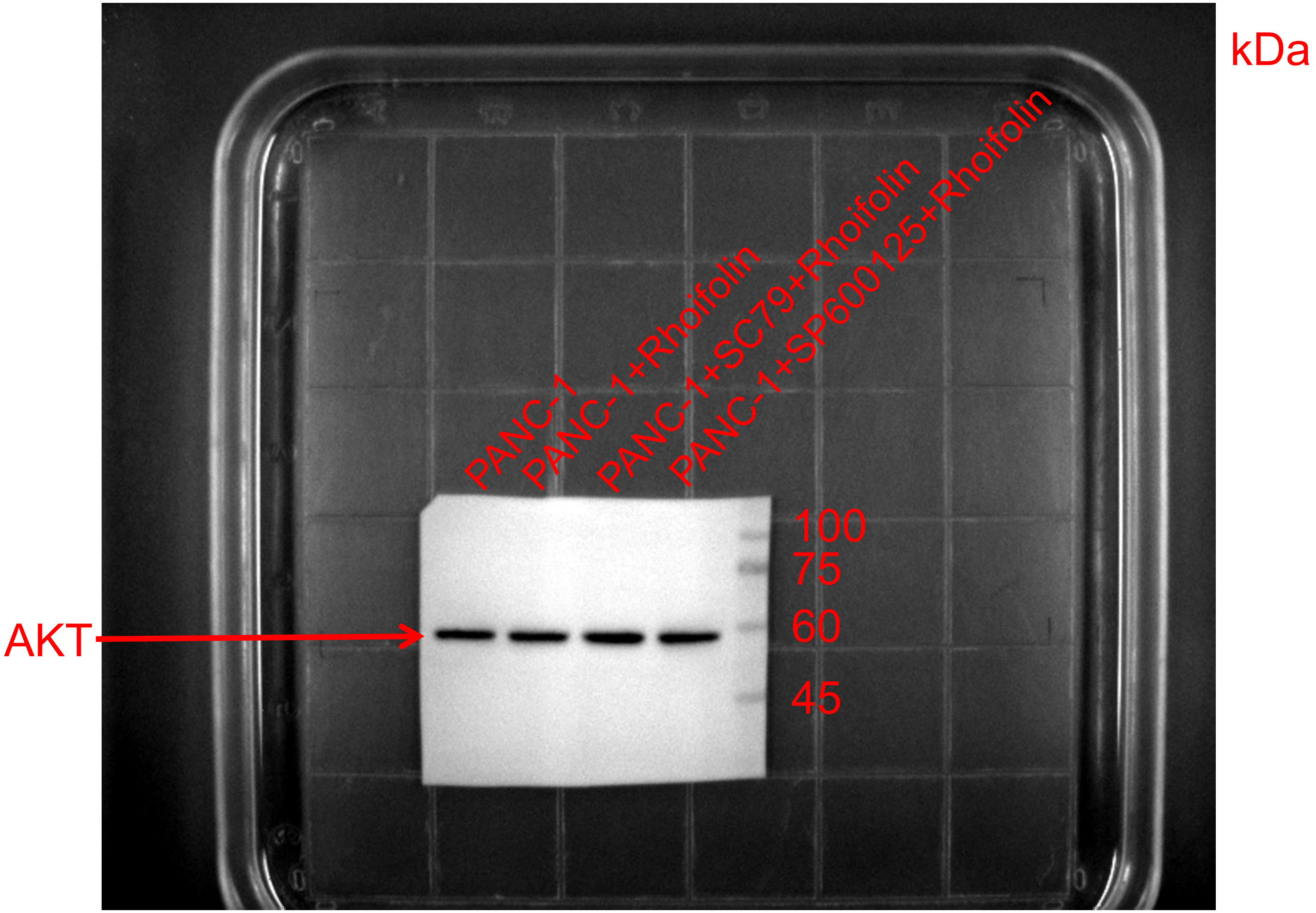

AKT, CST, 2920, 1:2000, 60kD; anti-Mouse IgG, Jackson, 115-035-003, 1:2000

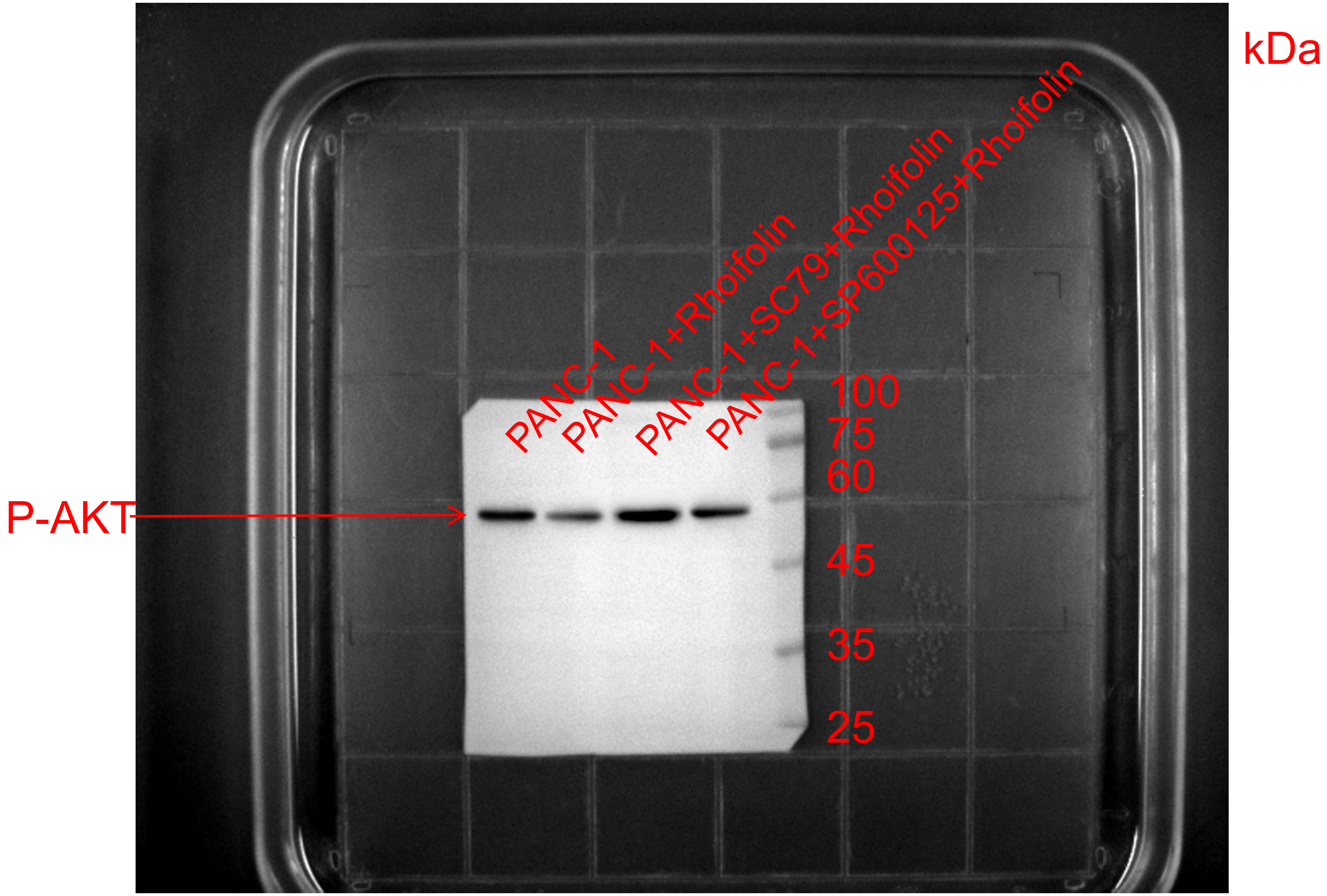

P-AKT, CST, 4060, 1:2000, 60kD;anti-Rabbit IgG, Jackson, 111-035-003, 1:2000
