# Supplementary material for: Rhoifolin from Plumula Nelumbinis exhibits anti-cancer effects in pancreatic cancer via AKT/JNK signaling pathways
Source: Sci Rep. 2022 Apr 5;12:5654. doi: 10.1038/s41598-022-09581-3 (PMC8983741; doi:10.1038/s41598-022-09581-3)
Supplement: Supplementary file 9 — Supplementary Legends. [file 41598_2022_9581_MOESM9_ESM.docx]

**Figure S1.** Analysis of gradient diluted vitexin. (A) Mass Spectrogram of vitexin. (B) Standard curve of vitexin. (C) Retention time of vitexin.

**Figure S2.** Analysis of gradient diluted rhoifolin. (A) Mass Spectrogram of rhoifolin. (B) Standard curve of rhoifolin. (C) Retention time of rhoifolin.

**Figure S3.** Analysis of gradient diluted apiin. (A) Mass Spectrogram of apiin. (B) Standard curve of apiin. (C) Retention time of apiin.

**Figure S4.** High performance liquid chromatography (HPLC) chromatograms of apiin, rhoifolin and vitexin with different digestive solutions.
